# Very long wave infrared quantum dot photodetector up to 18 μm

**DOI:** 10.1038/s41377-024-01436-y

**Published:** 2024-04-12

**Authors:** Xiaomeng Xue, Qun Hao, Menglu Chen

**Affiliations:** 1 School of Optics and Photonics, Beijing Institute of Technology, Beijing, 100081 China; 2 Westlake Institute for Optoelectronics, Fuyang, Hangzhou, 311421 China; 3 Physics Department, Changchun University of Science and Technology, Changchun, 130022 China

**Keywords:** Quantum dots, Imaging and sensing, Mid-infrared photonics, Photonic devices

## Abstract

Colloidal quantum dots (CQDs) are of interest for optoelectronic devices because of the possibility of high-throughput solution processing and the wide energy gap tunability from ultraviolet to infrared wavelengths. People may question about the upper limit on the CQD wavelength region. To date, although the CQD absorption already reaches terahertz, the practical photodetection wavelength is limited within mid-wave infrared. To figure out challenges on CQD photoresponse in longer wavelength, would reveal the ultimate property on these nanomaterials. What’s more, it motivates interest in bottom-up infrared photodetection with less than 10% cost compared with epitaxial growth semiconductor bulk. In this work, developing a re-growth method and ionic doping modification, we demonstrate photodetection up to 18 μm wavelength on HgTe CQD. At liquid nitrogen temperature, the responsivity reaches 0.3 A/W and 0.13 A/W, with specific detectivity 6.6 × 10^8^ Jones and 2.3 × 10^9^ Jones for 18 μm and 10 μm CQD photoconductors, respectively. This work is a step toward answering the general question on the CQD photodetection wavelength limitation.

## Abstract (web summary)

This work explores the boundary between nanocrystal and relative bulk, expanding the photoresponse wavelength limitation of colloidal quantum dot photodetector up to very long wave infrared.

## Graphical abstract

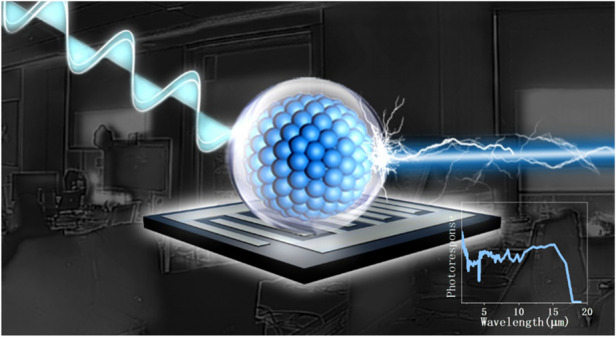

## Introduction

Infrared spectral range matching the atmospheric transparency region, is widely used in environmental monitoring, gas sensing and hazard detection. According to Rayleigh’s scattering law, scattering is inversely proportional to the fourth power of the wavelength. This means long wave infrared (LWIR, 6–15 μm) and very long wave infrared (VLWIR,15–30 μm) have obvious advantages on propagation distance. However, there are very few bulk semiconductors with such small enough band gaps. To date, the commercial infrared imaging devices based on those costly bulk and quantum-confined epitaxial materials^1^. For example, a single pixel 2.0–8.0 μm HgCdTe LWIR photodetector costs about 4000 euros, while the 2.0–10.6 μm HgCdTe LWIR photodetector costs about 5000 euros. The cost would further increase with extending infrared wavelength. Besides, epitaxial materials need complex flip-bonding process to couple with the read-out circuit (ROIC). As a result, the focal plane array (FPA) would cost even more. Colloidal quantum dots (CQDs) are of increasing interest due to the possibility of high-throughput solution processing^2–4^. The cost estimate for the infrared CQD film on the ROIC from related research^5^ is approximately 16 euros per FPA. The cost on the material itself would not obviously increase on longer infrared wavelength. What’s more, the CMOS compatibility makes it even a negligible component of the final camera cost for large-scale preparation.

The CQD spectral range is determined by the band gap of the respective compound, since the quantum confinement would increase the CQD band gap compared to the respective bulk material. As a result, only semimetal and narrow gap semiconductor are considered as the good choice for infrared wavelength^6^. Over the past several decades, there is great progress on CQD infrared photodetection, like infrared cameras^5,7,8^, upconversion photodetectors^9^, hyperspectral imagers^10^, bio-imaging^11^ and spectrometers^12^. Yet, most research focuses on near infrared^13^, short-wave infrared (SWIR)^14–18^ and mid-wave infrared (MWIR)^19–21^. Only very few CQD device could reach LWIR, however, the sensitivity drops quickly^22–24^. For example, in 2014, Keuleyan et al. reported the LWIR photodetection up to 12 μm^22^, with the responsivity 0.3 mA/W and the specific detectivity 6.5 × 10^6^ Jones. Intraband transition becomes an alternative for achieving LWIR, where the energy gaps inside the conduction band are naturally smaller than the relative interband transitions. In 2020, Ramiro et al.^23^ reported LWIR photodetection up to 9 μm *via* intraband transitions on PbS CQD, with typical responsivity ~10^–4^ A/W and the specific detectivity ~10^5^ Jones at 80 K. The 4-fold degeneracy in the PbS conduction band might contribute to a broadened spectrum and faster nonradiative relaxation. Recently, Zhang et al.^24^ achieved 8–12 μm photodetection utilizing intraband transition in HgTe CQD, which is 2 times smaller compared to the interband transition, with the typical responsivity ~10^−3^A/W and specific detectivity of ~10^7^ Jones at 80 K. The common low responsivity in LWIR CQD may be sign to the small ratio of the drift length to channel length and to the charge carrier generation efficiency. Previous analysis showed very short carrier lifetime on 12 μm LWIR CQD of ~10 ns at 80 K, 100 times worse compared to 5 μm MWIR CQD^25^. As a result, carriers barely travel over a few quantum dots given the moderate applied voltage and mobility.

Even though CQD absorption could reach terahertz^26,27^, expanding their photoresponse to LWIR and VLWIR is still challenging. On one hand, these wavelengths need very large size CQD even approaching Bohr radius^28^, where the colloidal stability is usually poor. On the other hand, such a small energy gap needs proper surface modification for precise doping control and long enough carrier drift length^29^. Yet, whether CQD photodetector could reach the VLWIR region has not been approved.

In this work, we demonstrate large-size HgTe CQD reaching LWIR and VLWIR by re-growth method. These CQDs show long-time stability up to several months in polar solution, providing a good platform for ligands exchange. Surface ionic modification is developed to obtain precise doping as well as surface passivation to reduce mercury emission. The carrier mobility is improved by a factor of 100, achieving 10 cm^2^/Vs, which improve the carrier transport efficiency. These treatments benefit 18 μm VLWIR CQD photodetector with responsivity of 0.3 A/W and specific detectivity of 6.6 × 10^8^ Jones at 80 K, as well as a 10 μm LWIR CQD photodetector with responsivity of 0.13 A/W and specific detectivity of 2.3 × 10^9^ Jones at 80 K. We also find that the responsivity is limited by the transport on photogenerated carriers as well as the low absorption coefficient.

## Results

For large-size HgTe CQD synthesis, excess mercury precursor is necessary, where Hg^2+^ would be served as surface ligands for good stability. In our case, the mercury-to-tellurium ratio is 4 to 1. Besides, re-growth method is developed with high reactive bis(trimethylsilyl)telluride (TMSTe) for rapid nucleation and low reactive tri-n-octylphosphine telluride (TOPTe) drop-by-drop for further nanocrystal growth. The flow chart of the synthesis process is shown in Fig. S1a.

**Fig. 1:**
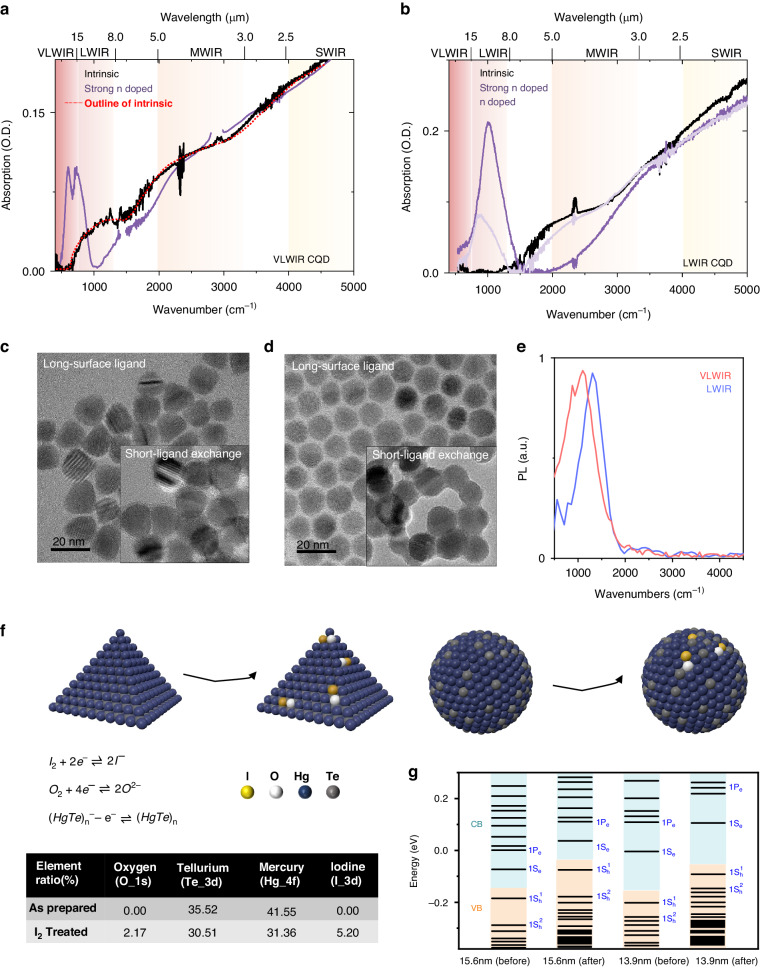
HgTe CQD. **a**, **b** Absorption spectra of VLWIR CQD and LWIR CQD with different doping at 300 K. **c**, **d** TEM of VLWIR and LWIR CQD dissolved in chlorobenzene solution, with the insert graph showing the CQD after ligand exchange dissolved in N,N-Dimethylformamide, respectively. **e** Photoluminescence spectra of I_2_ treated (intrinsic) VLWIR and LWIR CQD at 300 K. **f** A schematic diagram on as prepared and I_2_ treated tetrahedral and spherical CQD. The table shows the surface elements ratio on CQD before and after I_2_ treatment by XPS. **g** Calculated energy diagram on VLWIR and LWIR CQD before and after I_2_ treatment, respectively

The absorption spectra of obtained VLWIR and LWIR HgTe CQD are shown in Fig. 1a, b. Films are drop casted on ZnSe prism where the absorption is measured in total internal reflection. As prepared CQD solids are natural n doped from excess mercury, with obvious intraband transitions 1S_e_–1P_e_, peaked at 650 cm^−1^ and 1000 cm^−1^ for VLWIR and LWIR HgTe CQD, respectively. For VLWIR HgTe CQD, there is an obvious splitting the intraband transition. One peaked at 600 cm^−1^ with the other peaked at 745 cm^−1^, indicates a detailed energy structure in the 1P_e_ state due to the spin-orbital splitting. For LWIR HgTe CQD, this splitting is not observed which is probably from the CQD shapes and size dispersion. Figure 1c, d show the VLWIR and LWIR CQD shape and size determined by transmission electron microscope (TEM). The VLWIR CQD exhibit a near-tetrahedral shape with a diameter of 15.6 ± 1.4 nm, while the LWIR CQDs appear more spherical with diameter of 13.9 ± 1.4 nm. The corresponding histogram is presented in Fig. S1b, c. The shape difference is probably due to the TOPTe precursor concentration. As mentioned in the methods, following the same TMSTe nucleation step, lower TOPTe concentration with the same injection speed is used for larger size HgTe. The less reactive precursor produces the more anisotropic final particles. The TOPTe benefits the reduced energy tails^30^. However, TOP is not a good leaving group, likely binding to surface Hg sites as in the case of lead chalcogenides^31^. It hinders the long-time solution stability on large size CQDs. To facilitate the stable dispersion of the CQD without strong long-chain thiol ligands, mixed-phase ligand exchange^32^ is used with the process illustrated in Fig. S2. The TEM of CQDs after ligand exchange is shown in the inserted picture of Fig. 1c, d. The inter dot spacing is much reduced promoting the CQD electronic coupling and carrier transport efficiency^20^. As discussed in the later section, carrier mobility would be improved a factor of ~100.

Doping control is vital for high-performance infrared photodetection. Previous research shown that inducing S^2-^ in ligand exchange is an ideal oxidant for removing n type. However, there would be residue n type doping in this case even after long-time S^2-^ treatment. Iodine treatment is thereby developed. Avoiding the CQD solid collapse with high iodine concentration, 0.00002 M to 0.001 M iodine/ethanol solution are used. In addition, the band gap of HgI_2_ is 2.13eV^33^, a wide gap, which avoids the potential surface states. Iodine solutions of different concentrations and the corresponding absorption spectra are shown in Fig. S3. One could control the doping density by adjusting the iodine solution concentration. For example, the LWIR CQD solid treated with 0.00002 M iodine/ethanol solution, shows a bleached intraband transition and an induced interband transitions, the purple line in Fig. 1b. As the concentration increased, the intraband transitions are further reduced. With 0.0002 M iodine/ethanol solution for 1 min, the intraband transition just disappear, as shown in the black lines in Fig. 1a, b. We define the cutoff wavelength as 50% of the interband absorption edge. Before and after I_2_ treatment, the cutoff wavelength for interband transition shifts from 1250 cm^−1^ (8.0 µm) to 750 cm^−1^ (13 .0 µm) for VLWIR CQD solid, and from 2000 cm^−1^ (5.0 μm) to 1600 cm^−1^ (6.3 μm) for LWIR, respectively. Interestingly, one could see a strong overlap of intraband and interband transition in VLWIR CQD solids, while no obvious overlap is observed in LWIR, which agrees with our theoretical calculation discussed later. What’s more, the photoluminescence of intrinsic VLWIR and LWIR CQD is shown in Fig. 1e, peaked at 1025 cm^−1^ and 1320 cm^−1^, respectively. Both bands are blue-shifted compared to the absorption, opposite direction predicted by Stokes shift. The photoluminescence procedure is illustrated in Fig. S4. This may be assigned to thermal-activated peaks since the spectral response agrees with the absorption band edge as discussed in the later section.

To illustrate those optical features, we conduct morphology analysis like X-ray Diffraction (XRD) on the CQD phase composition and X-ray Photoelectron Spectroscopy (XPS) on the CQD surface elements, as well as a theoretical simulation on the detailed band structure. Fig. S5 shows that XRD results before and after I_2_ treatment are essentially identical, indicating the same lattice structure of *β*-HgTe (zinc-blende). The XPS analysis is shown in Fig. S6 with the element ratio table in Fig. 1f. Compared with the as prepared sample, the I_2_ treatment reduces the Hg to Te ratio from 1.17 to 1.03, introducing additional iodine (5.20%) and oxygen element (2.17%). The oxygen element may come from the residue air and water in the ethanol.

Based on the CQD shape, size, lattice structure and surface element ratio, we perform a theoretical simulation on detailed energy band, which is shown in Fig. 1g. DFT as implemented in the Vienna Ab initio simulation package (VASP) is used in all calculations, with details described in Fig. S7. There are some interesting features in the calculated bands. First, the iodine treatment shifts the absolute bands rather than change the energy band gaps, agreeing with the experiment. Second, the energy gap of interband transition is close to intraband transition on VLWIR CQDs, while there is 2 times difference on LWIR CQDs^24^. Third, the band gaps usually become narrower when the CQD radius increase. However, we notice the energy gaps between the first state and second state in the valence band become larger in the VLWIR CQD compared with LWIR CQD. This opposite trend in the valence band is also observed by tight binding calculations^34^. On the experimental side, spectra-electrochemistry^35^ might be useful to figure out these features. The less-dense valence band states may be helpful for low dark current and longer non-radiative lifetime^36^. In general, our theoretical calculation agrees with the experiment.

**Fig. 2:**
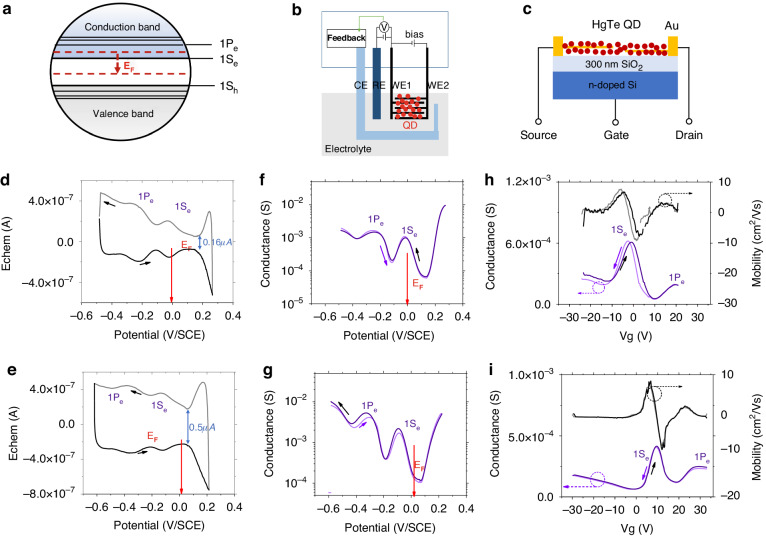
Transport characterization. **a** HgTe CQD Energy diagram. **b**, **c** Schematic diagram of electrochemistry and FET measurement, respectively. CE the counter electrode, RE the reference electrode, WE the working electrode. **d**, **e** Cyclic voltammetry on LWIR HgTe CQD before and after I_2_ treatment at 203 K, respectively. The arrows indicate forward and backward scan direction. Red arrows show the rest potential (Fermi Level) before the scan. **f**, **g** Conductance at different potential measured by electrochemistry with bi-potentiostat before and after I_2_ treatment, respectively. **h**, **i** FET transfer curve (purple lines) and differential mobility (black lines) at 80 K of HgTe CQD before and after I_2_ treatment, respectively

The energy diagram as well as transport properties are further determined by electrochemistry and field effect transistor (FET) measurement. Figure 2 shows the typical transport characterization on LWIR CQD before and after I_2_ treatment, while Fig. S8 shows the typical transport characterization on VLWIR CQD. Figure 2a–c show the schematic HgTe CQD energy diagrams, electrochemistry, and FET experiment. Agreeing with the absorption spectra, electrochemistry results show that as prepared CQD solids have electrons doped in the conduction band while the I_2_-treated CQD is near intrinsic, as shown in Fig. 2d, e, respectively. Typical electrochemistry provides Fermi level E_F_ by rest potential (red arrow) compared with saturated calomel electrode (SCE, −4.68 ± 0.02 eV/vacuum), as well as the absolute measurements of the filled and empty state energies with the application of liquid gate voltages (grey and black lines). The I_2_ treatment results a small positive 16 mV shift on Fermi level, and a negative 100 mV shift on CQD energy band. Since bi-potentiostat is used in electrochemistry, 5 mV bias difference is applied between the working electrodes where we obtain the conductance as a function of state density, as shown in Fig. 2f, g. Both cyclic voltammetry and conductance results show the quantum-confined electronic states, noted as 1Se and 1Pe. Additionally, compared with the as prepared CQD solid, the reversible voltammetry currents increase a factor of 2–3 after I_2_ treatment, which may come from the redox process of I_2_/I_3_^−^/I^−^. Fig. S9 shows the cyclic voltammetry measurement on 0.001 M I_2_/ethanol. Iodine oxidation clearly occurs in two steps, attributing to the I_2_/I_3_^–^ and I_3_^–^/ I^–^ processes at lower and higher potentials, respectively. The increased voltammetry currents mean more external carrier injection or depletion is needed to tune doping, which is beneficial for doping stabilization in these CQD solids.

The doping density in as prepared CQD is 0.92 ± 0.05 e/dot, estimated by integrating the charging current in cyclic voltammetry^35^. The doping densities are further approved by FET shown in Fig. 2h, i. Following the conventions used for the gate voltage, holes have a negative differential mobility while electrons have the positive one. Here, although as prepared CQD solid shows negative trans-conductance near 0 V gate potential, they are n doped. The negative trans-conductance is from the Pauli blockade in the carrier density more than half filled 1Se state. Then, the dip in the transfer curve appears when 1Se state is fully filled where the peak means half filling. There is also a shoulder in Fig. 2h at positive gate potential, which is 1Pe state. The carrier density is 1.2 ± 0.1 e/dot in as prepared CQD solid determined by FET, following the same calculation process, which is slightly larger than electrochemistry probably due to different surrounding environments. Both electrochemistry and FET show I_2_ treated CQD solid almost intrinsic (slightly *p* type, ≤0.05 h/dot).

The differential mobility is extracted, black and gray lines in Fig. 2h, i. The mobility could reach as high as 10 cm^2^/Vs in the linear region, a factor of 100 larger compared to CQD solid without mixed phase ligand exchange, shown in Fig. S10. This agrees to the reduced spacing between CQD as shown by the TEM. Besides, the additional iodine and oxygen element on the CQD surface do not affect carrier transport efficiency and the CQD coupling. The VLWIR CQD solids show similar features in transport characterization. Although with denser states in the conduction band, the 1Pe state is less obvious. The mobilities have a weak negative temperature dependence from 6 cm^2^/Vs to 10 cm^2^/Vs for 300 to 80 K, in Fig. S11.

## Discussion

**Fig. 3:**
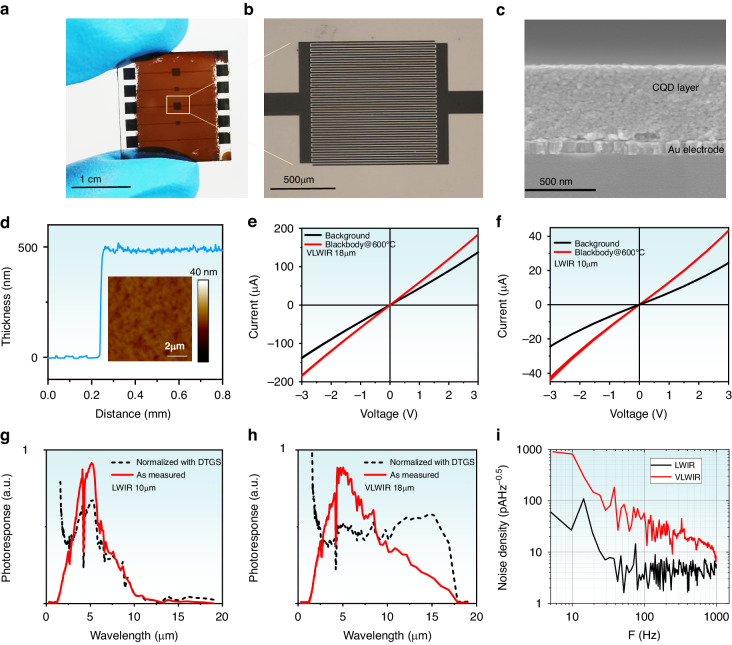
HgTe CQD Photodetectors. **a, b** The real picture of the photodetector. **c** SEM cross-section picture. **d** Thickness measured by step meter and surface roughness mapping by AFM. **e, f** I-V curves characterization on VLWIR and LWIR CQD photoconductor at 80 K. **g, h** Spectral response of VLWIR and LWIR CQD photoconductor at 80 K, respectively. The red lines show as measured response spectra. The black dash lines are normalized response spectra with DTGS. **i** Noise spectra on VLWIR and LWIR CQD photoconductor at 80 K with 3 V bias, respectively

Both VLWIR and LWIR CQD photoconductors are demonstrated shown in Fig. 3. CQDs are spin-coated on Al_2_O_3_ substrate with 25 pairs of Au interdigit electrode, whose length is 1 mm, gap 10 um, width 10 um. After ethanedithiol (EDT)/HCl ligand exchange^37^, the solids are soaked in iodine/ethanol solution for 1 min. Then, the solids would be rinsed with isopropanol and dried with N_2_ gas. Figure 3a–c show the real picture of the photodetector, electrodes, as well as scanning electron microscope (SEM) cross-section picture. Figure 3d shows the thickness measured by step meter, as well as the surface roughness mapping by atomic force microscope (AFM). Both SEM and AFM show the solid thickness is ~500 nm with high uniformity. Figure 3e, f show the photocurrent and dark current curves on VLWIR and LWIR CQD photodetector, respectively. The infrared radiation is from the 600 °C blackbody. Cooled with liquid nitrogen, the VLWIR CQD photoconductor with 3 V bias shows 47.8 μA photocurrent, 136 μA dark current, which is 0.35 on/off (photocurrent to dark current) ratio. Under the same condition, the LWIR CQD photoconductor exhibits 18.2 μA photocurrent, 24.1 μA dark current and 0.76 on/off ratio. The higher on/off ratio in LWIR CQD photoconductor compared with VLWIR, comes from the larger energy gap and lower thermal activated carrier densities. Figure 2g, h show the spectral response at liquid nitrogen temperature before and after normalization with DTGS. VLWIR CQD photoconductor shows photoresponse up to 18 μm (550 cm^−1^). LWIR CQD photoconductor shows photoresponse up to 10 μm (1000 cm^−1^). The spectral response with wavenumber as the x-axe is provided in the Fig. S12. Obviously, there is a red shift on photoresponse band edge compared with relative interband absorption at room temperature. This is from the band shift of the relative bulk material as a function of temperature. For LWIR, we successfully measure spectral response from 300 K to 80 K, in Fig. S13, where the room temperature spectral response edge is 1720 cm^−1^ (5.9 μm), comparable with the room temperature absorption edge 1600 cm^−1^ (6.3 μm). The red shift is ~700 cm^−1^ with the temperature cooling from 300 K to 80 K. This shift value is larger than MWIR HgTe CQD^25,37^, which is ~400–500 cm^−1^. Additionally, as shown in Fig. S13, this redshift, rather than a simple translation on the band edge, looks like a new state gradually rising in the LWIR region. The LWIR CQD photoconductor shows clear quantum confined states like MWIR CQD photoconductor as shown in Fig. S14, while the normalized spectral response on VLWIR CQD photoconductor is more like bulk semiconductor, where no obvious quantum state could be figured out.

The photodetection properties are further investigated. Responsivity (${\mathscr{R}}$) and specific detectivity (${D}^{* }$) are two typical physics quantities reflecting the photodetector performance. ${\mathscr{R}}=\frac{{I}_{ph}}{P}$, where *I*_*ph*_ is photocurrent, *P* incident power. The incident power from the 600 °C blackbody light source is 319 μW/mm^2^ and 289 μW/mm^2^, corresponding to 10 μm and 18 μm wavelength, respectively. The effective detector area A is 0.5 mm^2^. As a result, the responsivity is 0.3 A/W and 0.13 A/W on VLWIR and LWIR CQD photoconductor at 80 K, respectively. The external quantum efficiency ${\rm{EQE}}=\frac{1.24{\mathscr{R}}}{\lambda \left. ({\rm{\mu }}{\rm{m}}\right)}$ is 2% and 1.6% on VLWIR and LWIR CQD photoconductor at 80 K, respectively. The values are comparable with MWIR CQD photoconductor whose EQE is 5% under the similar conditions^25^. Since the optical absorption value is small ~5% on the 500 nm thickness LWIR CQD solid, Fig. S15a, the internal quantum efficiency (IQE) is 40% and 30% on VLWIR and LWIR CQD photoconductor at 80 K, respectively. The absorption coefficient $\alpha =\frac{4\pi k}{\lambda }$, reach ~800 cm^−1^, where k is the extinction coefficient. The absorption coefficient value is one order smaller than MWIR CQD with mixed phase ligands exchange with the value 10^4 ^cm^−1^ and SWIR CQD with the value ~ 6000 cm^−1^, Fig. S15b. This may not simply assign to the larger CQD size and lower CQD density^38^ discussed previously. The sparse hole density may be another reason. Because of the low absorption coefficient, thicker film is necessary for ideal EQE. However, the carrier drift length is another concern.

We further investigate the effect from carrier mobility on ${\mathscr{R}}$ and EQE. The near intrinsic low carrier mobility (~0.1 cm^2^/Vs) LWIR CQD photoconductor show *R* = 0.2 mA/W and EQE = 0.025%, a factor of 670 worse compared with the high mobility (~10 cm^2^/Vs) LWIR CQD photoconductor. The difference in responsivity is much larger compared with the mobility difference which is about 100 times. This may due to the stronger carrier scattering and shorter drift length in the low mobility CQD solid, as discussed below.

The EQE is proportional to the ratio between the carrier drift length and the electrode gap. Following the reference model^29^, the responsivity could be expressed as ${\mathscr{R}}=\eta \tau ({\mu }_{e}+{\mu }_{h})\frac{{eV\; L}}{{{hv\; L}}_{{gap}}}$, *η* exciton ionization probability, *τ* carrier lifetime, ${\mu }_{e}$ electron mobility, ${\mu }_{h}$ hole mobility, ${hv}$ the input photon energy, *L*_*gap*_ = 10 μm electrode gap, V = 3 V applied voltage, L = 50 mm total electrode length. The maximum value on *η* is one, allowing the lower limit estimation on carrier lifetime *τ*. For VLWIR CQD, the *τ* is 15 ns for 10 cm^2^/Vs high mobility sample and 2.2 ns for 0.1 cm^2^/Vs low mobility sample, respectively. The transit time ${\tau }_{t}=\frac{{{L}_{{gap}}}^{2}}{\mu V}$, the value is 32 ns for 10 cm^2^/Vs high mobility sample, 3.2 μs for 0.1 cm^2^/Vs low mobility sample, respectively. The hopping time ${\tau }_{{hop}}$, which is estimated by mobility *μ* from Einstein’s relations on diffusion in three dimension^39^ ${\tau }_{{hop}}=\frac{e{d}^{2}}{6\mu {k}_{b}T}$, where *d* is the CQD diameter, ${k}_{b}$ Boltzmann constant, *T* temperature. With VLWIR CQD diameter ~15.6 nm, the hopping time ${\tau }_{{hop}}$ is several picoseconds for a mobility of 10 cm^2^/Vs, and near nanoseconds for a mobility of 0.1 cm^2^/Vs. Both transit time ${\tau }_{t}$ and carrier lifetime *τ* are much longer than ${\tau }_{{hop}}$ in high mobility CQD solid. The carrier lifetime *τ* is comparable with hopping time in low mobility CQD solid.

The carrier drift length L_drift_ = μ(V/L_gap_)τ, the value is $4.5\times {10}^{-6}$ m for 10 cm^2^/Vs high mobility sample. The ratio between the carrier drift length and the electrode gap is 45% in the high mobility VLWIR CQD and 0.066% in the low mobility VLWIR CQD. The ratio is comparable with the relative IQE. These results also indicate that our proper ligands modification successfully achieve high mobility and decent carrier lifetime. This is vital for photodetection in LWIR. Still, the carrier lifetime is shorter compared with HgCdTe^40^ and InAs/InAsSb^41^ type-II superlattices with similar band gaps, as well as the theoretical prediction in HgTe CQD^36^. Further investigation is needed.

The specific detectivity ${D}^{* }=\sqrt{{\rm{A}}}\frac{{I}_{{ph}}}{{I}_{n}P}$, where ${I}_{n}$ is the measured noise density. In our photoconductor device, the dominate noise comes from 1/f noise due to carrier density and mobility fluctuations, as well as shot noise generated in the photon generation-recombination process, shown in Fig. 3i. Low frequency 1/f noise below several-ten Hz may from the set up like the amplifier, rather than the photodetector itself. On LWIR CQD photoconductor at 80 K with 3 V bias, the dominate measured noise (black line) at 500 Hz is 4 pA Hz^−1/2^, close to the theoretical shot noise ${I}_{{shot}}=\sqrt{4e{I}_{d}\Delta f}$ = 2.7 pAHz^−1/2^, where *e* is elementary charge (1.6 × 10^−19^C), ${I}_{d}$ the dark current, ∆*f* the band width. On VLWIR CQD photoconductor, the measured noise (red line) is mainly from 1/f noise, which is 32 pAHz^−1/2^ at 500 Hz, while the theoretical shot noise is 9.4 pAHz^−1/2^. With the measured noise density at 500 Hz, the detectivity s is 6.6 × 10^8^ Jones and 2.3 × 10^9^ Jones on VLWIR and LWIR photoconductor at 80 K, respectively.

The Background limited (BLIP) detectivity is ${D}_{{BLIP}}^{* }(\lambda ,f)=\frac{\lambda }{2{hc}}({\frac{\eta }{{Q}_{b}})}^{1/2}$, where the *λ* is wavelength, h Planck’s constant, *c* speed of light, *η* quantum efficiency, *Q*_*b*_ the 300 K blackbody integrated flux from the specified wavelength to shorter wavelengths over a solid angle of 2π. From this formula, we calculate that BLIP detectivity at 10 μm and 18 μm is 3.3 × 10^10^ Jones and 3.25 × 10^10^ Jones, respectively. For the real devices, transmittance of the electrodes and CQD solid absorption should also be considered. The BLIP detectivity of the device could be calculated as ${D}_{{device},{BLIP}}^{* }=\sqrt{{Trans}\times {Abs}}{D}_{{BLIP}}^{* }$. Trans is the transmittance of gold electrodes, which is 100% in the photoconductor since the light is directly incident on the CQD film. *Abs* is absorption value. Currently, Abs is only 5% in this device. There is potential for high sensitivity LWIR and VLWIR CQD photodetectors. The related performance parameters of LWIR and VLWIR devices are shown in Table 1. We also compare the typical LWIR/VLWIR photodetectors in Table S1.

**Table 1:** Data for LWIR and VLWIR detectors

Device/Formula	Wavelength μm	Bias V	T K	D* Jones	${\mathfrak{R}}$ AW^−1^	EQE %	IQE %	E_a_ (Activation energy)
Formula				$\sqrt{{\rm{A}}}\frac{{I}_{{ph}}}{{I}_{n}P}$	$\frac{{I}_{ph}}{P}$	$\frac{1.24{\mathscr{R}}}{\lambda \left. ({\rm{\mu }}{\rm{m}}\right)}$	$\frac{{I}_{{ph}}/e}{{\varphi }_{{abs}}}$	exp(-E_a_/k_B_T)
LWIR	10	3	80	2.3 × 10^9^	0.13	1.6	30	68
VLWIR	18	3	80	6.6 × 10^8^	0.3	2	40	40

**Fig. 4:**
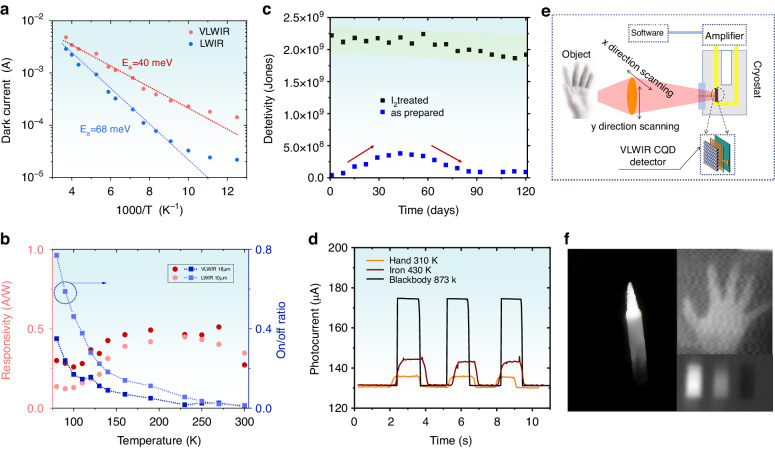
Photodetection property. **a** Dark currents on LWIR and VLWIR CQD devices at 3 V bias as function of the reciprocal of temperature, respectively. **b** The responsivity and pure photocurrent to dark current ratio (on/off ratio) as function of temperature on VLWIR and LWIR CQD devices, respectively. **c** Detectivity variation with time on LWIR CQD device with (black) and without (blue) I_2_-treatment. **d** The temporal response of LWIR CQD device, exposed to objects with different temperatures. **e, f** Imaging process and thermal imaging by VLWIR CQD device

Stronger 1/f noise shown in VLWIR CQD solid may come from the high thermal carrier density risen from the small energy gaps. We investigate the dark current as a function of temperature to extract activation energy on thermal carriers, as shown in Fig. 4a. The activation energy E_a_ is 40 mV and 68 mV on VLWIR (red) and LWIR (blue) CQD solids, fitting by exp(-E_a_/k_B_T) with k_B_ the Boltzmann constant. The activation energy E_a_ is comparable with 50% of the interband energy gap on VLWIR and LWIR CQD. Fig. S16 shows dark current as a function of applied bias at different temperatures. Figure 4b shows the trend on responsivity (red) and pure photocurrent to dark current ratio (blue) with temperature on VLWIR and LWIR CQD photodetector. The ratio is monotonous with temperature, reaching maximum value 0.34 and 0.76 at 80 K on VLWIR and LWIR CQD photodetector, respectively. At 300 K, the minimum value on/off ratio is 0.008 and 0.014 on VLWIR and LWIR CQD photodetector, respectively. The responsivity is non-monotonic with temperature, where the variation is small. The long-time stability on CQD photodetector is also investigated. Without any encapsulation, the LWIR CQD photoconductor after I_2_ treatment could maintain D* above 2 × 10^9^ Jones after four months exposed to air, as shown in Fig. 4c. Meanwhile, without I_2_ treatment, the detectivity of LWIR CQD photoconductor, which is stored in ambient environment would first increase than decrease. The increasing in D* is due to the gradual oxidation in air, which removing the n-type doping on the CQD. However, the oxidation is uncontrollable, where the further oxidation hinders the device performance. The I_2_ treatment, may contribute to a dense cover layer on the surface. Figure 4d shows the temporal response of VLWIR CQD detector when exposed to human hand, heated iron and 600 °C blackbody, where the photocurrents are 5.8 μA, 12.7 μA and 47.8 μA, respectively. The one-pixel imaging process is illustrated by Fig. 4e. The image of iron is shown in Fig. 4f (left). The thermal information on the human hand and different temperature water (80 °C, 50 °C, 18 °C, from left to right) is shown in Fig. 4f (right), various gray levels reflect different object temperatures.

In this work, we investigate large-size HgTe CQD and their photodetection properties in LWIR and VLWIR region. Large-size HgTe CQD up to 15.6 nm in diameter are synthesized by re-growth method. Iodine treatment on CQD solids precisely tunes doping from strong n type to near intrinsic, as well as surface passivation to reduce further oxidation in the ambient environment. Besides, the mix-phase ligands exchange process could improve carrier mobility a factor of 100, achieving 10 cm^2^/Vs, which improve the carrier transport efficiency. These treatments benefit 18 μm VLWIR CQD photodetector with responsivity of 0.3 A/W and specific detectivity of 6.6 × 10^8^ Jones at 80 K, as well as a 10 μm LWIR CQD photodetector with responsivity of 0.13 A/W and specific detectivity of 2.3 × 10^9^ Jones at 80 K. The responsivity in high mobility VLWIR CQD photoconductor is a factor of 670 larger than the low mobility reference sample. We find that the responsivity is limited by the ratio between the carrier drift length and the electrode gap, as well as the absorption coefficient. These results also indicate that proper ligands modification to achieve high mobility and decent carrier lifetime, is vital for long-wavelength photodetection. This work not only expands the photodetection wavelength on CQD, but also motivates interest in bottom-up infrared photodetection beyond costly epitaxial growth semiconductor bulk.

## Materials and Methods

### Synthesis

#### LWIR CQD

For mercury precursor, 108 mg HgCl_2_ (0.4 mmol) in 8 mL oleylamine (OAm) is stirred at 100 °C until it turns into transparent solution. For, tellurium precusor, 21 μL (0.075 mmol) bis(trimethylsilyl)telluride (TMSTe, Fisher, 98%) is diluted in 900 μL degassed OAm. At 120 °C, the TMSTe solution is injected into the mercury precursor rapidly with reaction for 1 min. Then 0.02 M trioctylphosphine telluride (TOPTe) solution is injected at a constant speed of 0.5 mL/min for 2.5 min. Then, the solution is cooled with running water.

#### VLWIR CQD

All steps are the same as LWIR CQD, expect that the TOPTe concentration is 0.01 M with injection speed 0.5 mL/min for 5 min.

#### Cleaning process

Precipitate the CQD by adding acetone until the solution is turbid. After centrifugation, black precipitate is collected and washed with acetone again to remove excess OAM. Then the precipitate is re-dispersed in 2 mL hexane.

#### Mixed phase ligand exchange

The process mainly follows previous work^34^. The difference is that no Hg^2+^ or S^2-^ is added in liquid ligand exchange since the doping adjustment is done after solid ligand exchange with EDT/HCl/IPA.

#### Iodine treatment

Typically, the CQD solids are soaked in 2 × 10^−4 ^M iodine/ethanol solution for 1 min for near intrinsic doping.

### Morphology characterization

#### XRD

The measurement is performed with the D8 ADVANCE X-ray diffractometer from Bruker, Germany. The instrument was operated with a tube current of 40 mA and a tube voltage of 40 kV. The Cu target had a wavelength of 1.5406 Å, while the Co target had a wavelength of 1.79026 Å.

#### XPS

XPS analysis is done with PHI QUANTERA-II SXM, by ULVAC-PHI Corporation, Japan. The X-ray source is AlKa (Al target, 1486.6 eV).

### Optical characterization

#### Absorption

FTIR (FOLI20, Ying Sa Optical Instruments) is used. Films are drop casted on ZnSe prism where the absorption is measured in total internal reflection, or ZnSe window for direct absorption measurement. Gaussian fittings on spectrum are shown in Fig. S17.

#### Photoluminescence

Photoluminescence is measured by homemade set up with details described in supporting information.

### Transport characterization

#### Electrochemistry

The measurement mainly follows the reference^34^. CQD solid is prepared on Au interdigitated electrodes on glass substrate with spacing d = 15μm and finger width d_0_ = 15 μm. The electrolyte is 0.1 M anhydrous tetrabutylammonium perchlorate/ propylene carbonate cooled in an ethanol/dry ice bath at 203 K. The bi-potentiostat model is PalmSens4.

#### Field effect transistor

Keithley 2636 A source meter is used to collect data. The substrates are Silicon wafers with 300 nm SiO_2_. The electrode design is mentioned in the main text as the photoconductor where 25 pairs of interdigitated evaporated gold electrodes with finger width 10 microns, gap 10 microns (channel length in FET), and finger length 1 mm (channel width in FET). Details are described in the supporting information. Table S2 also summarizes the transport property on both high and low mobility CQD solids.

### Photodetection

#### Spectral response

CQD Photodetector is connected to FTIR (FOLI20, Ying Sa Optical Instruments) as an external detector for spectral response.

#### Noise spectra

The noise is amplified then collected by noise analyzer SR770, from Stanford Research Systems.

#### I-V curves

The CQD photodetectors are shined with 600 °C blackbody radiation from HT-P1000 radiation source, Shanghai Yun Jian Intelligent Technology, while the I-V curves are collected by Keithley 2602B source meter.

#### Single pixel imaging

Linear motorized stages are used in the scanning process. An infrared lens with focal length 30 mm is used. With 3 kHz sampling rate, the photocurrent is recorded and used to construct images.

#### Temporal response

The schematic diagram of the response speed measurement is shown in Fig. S18a. The light emitted by the laser fiber enters the polarization controller, which controls the polarization state of the light. Then, the light entering the electro-optical modulator is modulated into a pulsed laser signal. The signal waveform generator is connected to the electro-optical modulator, adjusting the parameters such as frequency and amplitude of laser signal. Meanwhile, the signal waveform generator is connected to the oscilloscope to observe the modulated signal, which is then incident on the photodetector.

The photodetector is connected in series with a bias voltage and a resistor that matches the internal resistance value of the detector. The time constant rise (${{\rm{\tau }}}_{{\rm{rise}}}$) refer to the time required for the signal voltage to rise from 10% of the maximum value to 90%. The response speed of LWIR and VLWIR detectors are shown in Fig. S18b, c. The ${{\rm{\tau }}}_{{\rm{rise}}}$ are 93.4 μs and 368 μs respectively.

### Supporting information available

SI includes details on synthesis process and size distribution of CQD, mixed phase ligands exchange process, iodine treatment, photoluminescence, X-ray Diffraction, X-ray Photoelectron Spectroscopy, theoretical band calculations, typical transport characterization on VLWIR HgTe CQD, electrochemistry on iodine solution, transport on CQD without mixed phase ligands exchange, mobility as a function of temperature, the spectral response with wavenumber as the x-axe, the spectral response on LWIR CQD at different temperatures, the spectral response comparison, coefficient calculation and dark current at different temperatures, gaussian fittings on spectrum, detectivity comparison and temporal response, transport property summary, spectral detectivity.

### Supplementary information

Supplemental materials for publication

## References

[1] Rogalski, A. Infrared Detectors. 2nd edn. (Boca Raton: CRC Pssress, 2010).

[2] Kagan CR, Lifshitz E, Sargent EH, Talapin DV (2016). Building devices from colloidal quantum dots. Science.

[3] Liu M (2021). Colloidal quantum dot electronics. Nat. Electron..

[4] Huang P (2023). Nonlocal interaction enhanced biexciton emission in large CsPbBr_3_: nanocrystals. elight.

[5] Greboval C (2022). Photoconductive focal plane array based on HgTe quantum dots for fast and cost-effective short-wave infrared imaging. Nanoscale.

[6] Lu H, Carroll GM, Neale NR, Beard MC (2019). Infrared Quantum Dots: Progress, Challenges, and Opportunities. ACS Nano.

[7] Liu J (2022). A near-infrared colloidal quantum dot imager with monolithically integrated readout circuitry. Nat. Electron..

[8] Zhang S (2023). Wafer-scale fabrication of CMOS-compatible trapping-mode infrared imagers with colloidal quantum dots. ACS Photonics.

[9] Zhou W (2020). Solution-processed upconversion photodetectors based on quantum dots. Nat. Electron..

[10] Tang X, Ackerman MM, Guyot‐Sionnest P (2019). Acquisition of hyperspectral data with colloidal quantum dots. Laser Photonics Rev..

[11] Bruns OT (2017). Next-generation in vivo optical imaging with short-wave infrared quantum dots. Nat. Biomed. Eng..

[12] Grotevent MJ (2022). Integrated photodetectors for compact Fourier-transform waveguide spectrometers. Nat. Photonics.

[13] Sun B (2022). Fast near-infrared photodetection using III-V colloidal quantum dots. Adv. Mater..

[14] Rogach A (1999). Colloidally prepared HgTe nanocrystals with strong room-temperature infrared luminescence. Adv. Mater..

[15] Chen M (2014). Photocurrent enhancement of HgTe quantum dot photodiodes by plasmonic gold nanorod structures. ACS nano.

[16] Geiregat P (2018). Continuous-wave infrared optical gain and amplified spontaneous emission at ultralow threshold by colloidal HgTe quantum dots. Nat. Mater..

[17] Yang J (2022). Ligand-engineered HgTe colloidal quantum dot solids for infrared photodetectors. Nano Lett..

[18] Dang TH (2023). Bias reconfigurable photoresponse of an infrared nanocrystal film integrated into a coupled fabry-perot resonator. ACS Photonics.

[19] Keuleyan S, Lhuillier E, Brajuskovic V, Guyot-Sionnest P (2011). Mid-infrared HgTe colloidal quantum dot photodetectors. Nat. Photonics.

[20] Lan X (2020). Quantum dot solids showing state-resolved band-like transport. Nat. Mater..

[21] Dang TH (2023). Multiresonant grating to replace transparent conductive oxide electrode for bias selected filtering of infrared photoresponse. Nano Lett..

[22] Keuleyan SE, Guyot-Sionnest P, Delerue C, Allan G (2014). Mercury telluride colloidal quantum dots: electronic structure, size-dependent spectra, and photocurrent detection up to 12 μm. ACS Nano.

[23] Ramiro I (2020). Mid- and Long-Wave infrared optoelectronics via intraband transitions in PbS colloidal quantum dots. Nano Lett..

[24] Zhang H, Peterson JC, Guyot-Sionnest P (2023). Intraband transition of HgTe nanocrystals for Long-Wave infrared detection at 12 μm. ACS Nano.

[25] Chen M (2019). High carrier mobility in HgTe quantum dot solids improves Mid-IR photodetectors. ACS Photonics.

[26] Lhuillier E (2016). Infrared photodetection based on colloidal quantum-dot films with high mobility and optical absorption up to THz. Nano Lett..

[27] Goubet N (2018). Terahertz HgTe nanocrystals: beyond confinement. J. Am. Chem. Soc..

[28] Talapin DV, Lee JS, Kovalenko MV, Shevchenko EV (2010). Prospects of colloidal nanocrystals for electronic and optoelectronic applications. Chem. Rev..

[29] Guyot-Sionnest P, Peterson JC, Melnychuk C (2022). Extracting bulk-like semiconductor parameters from the characterization of colloidal quantum dot film photoconductors. J. Phys. Chem. C..

[30] Zhang H, Guyot-Sionnest P (2020). Shape-Controlled HgTe colloidal quantum dots and reduced spin-orbit splitting in the tetrahedral shape. J. Phys. Chem. C..

[31] Moreels I (2011). Size-tunable, bright, and stable PbS quantum dots: A surface chemistry study. ACS Nano.

[32] Xue X (2023). High-operating-temperature mid-infrared photodetectors via quantum dot gradient homojunction. Light Sci. Appl..

[33] Solanki, Kashyap A, Nautiyal T, Auluck S (1997). Band structure and optical properties of HgI_2_. Phys. Rev. B.

[34] Allan G, Delerue C (2012). Tight-binding calculations of the optical properties of HgTe nanocrystals. Phys. Rev. B.

[35] Chen M, Hao Q, Luo Y, Tang X (2022). Mid-infrared intraband photodetector via high carrier mobility HgSe colloidal quantum dots. ACS Nano.

[36] Melnychuk C, Guyot-Sionnest P (2022). Thermodynamic limits to HgTe quantum dot infrared detector performance. J. Electron. Mater..

[37] Chen M (2023). Universal homojunction design for colloidal quantum dot infrared photodetectors. Adv. Mater. Technol..

[38] Chehaibou B (2023). Modeling HgTe complex optical index from bulk to nanocrystal layers. J. Phys. Chem. C..

[39] Guyot-Sionnest P (2012). Electrical transport in colloidal quantum dot films. J. Phys. Chem. Lett..

[40] Wen H, Pinkie B, Bellotti E (2015). Direct and phonon-assisted indirect auger and radiative recombination lifetime in HgCdTe, InAsSb, and InGaAs computed using Green’s function formalism. J. Appl. Phys..

[41] Olson BV (2013). Identification of dominant recombination mechanisms in narrow-bandgap InAs/InAsSb type-II superlattices and InAsSb alloys. Appl. Phys. Lett..

