# Supplementary material for: Very long wave infrared quantum dot photodetector up to 18 μm
Source: Light Sci Appl. 2024 Apr 12;13:89. doi: 10.1038/s41377-024-01436-y (PMC11014860; doi:10.1038/s41377-024-01436-y)
Supplement: Supplementary file 1 — Supplemental materials for publication [file 41377_2024_1436_MOESM1_ESM.docx]

**Supporting Information**

**Very Long Wave Infrared Quantum Dot Photodetector up to 18 μm**

Xiaomeng Xue^1,2#^, Qun Hao^3,1#^, Menglu Chen^1,2,3#^*

1. School of Optics and Photonics, Beijing Institute of Technology, Beijing 100081, China
2. Westlake Institute for Optoelectronics, Fuyang, Hangzhou 311421, China
3. Physics Department, Changchun University of Science and Technology, Changchun 130022, China

^#^These authors contributed equally to this work

*Corresponding author：[menglu@bit.edu.cn](mailto:menglu@bit.edu.cn)

**Content**

1. **Synthesis process and size distribution of CQD.**
2. **Mixed phase Ligands exchange process.**
3. **Iodine treatment.**
4. **Photoluminescence.**
5. **X-ray Diffraction.**
6. **X-ray Photoelectron Spectroscopy.**
7. **Theoretical band calculations**
8. **Typical transport characterization on VLWIR HgTe CQD.**
9. **Electrochemistry on iodine solution.**
10. **Transport on CQD without mixed phase ligands exchange.**
11. **Mobility as a function of temperature.**
12. **The spectral response with wavenumber as the x-axe.**
13. **The spectral response on LWIR CQD at different temperatures.**
14. **The spectral response comparison.**
15. **Absorption coefficient calculation.**
16. **Dark current at different temperatures.**
17. **Gaussian fittings on spectrum**
18. **Detectivity comparison**
19. **Temporal response**
20. **Transport property summary**
21. **Spectral Detectivity**
22. **Synthesis process and size distribution of CQD.**

LWIR CQD: For mercury precursor, 108 mg HgCl_2_ (0.4 mmol) in 8 mL oleylamine (OAm) is stirred at 100 ℃ until it turns into transparent solution. For, tellurium precusor, 21 μL (0.075 mmol) bis(trimethylsilyl)telluride (TMSTe, Fisher, 98%) is diluted in 900 μL degassed OAm. At 120°C, TMSTe solution is firstly injected rapidly into the mercury precursor with reaction for 1 min. 0.02 M trioctylphosphine telluride (TOPTe) solution is injected at a constant speed of 0.5 mL/min for 2.5 min. After that, the solution is cooled with running water.

VLWIR CQD：All steps are the same as LWIR CQD, expect that the TOPTe concentration is 0.01M with injection speed 0.5 ml/min for 5 min.

Synthesis flow chart is shown in **Figure S1a**. **Figure S1b-1c** show the VLWIR and LWIR CQD shape and size determined by transmission electron microscope (TEM), where VLWIR CQD is near tetrahedral with diameter of 15.6 ±1.4 nm and LWIR CQD more spherical with diameter of 13.9 ±1.4 nm with the histogram.


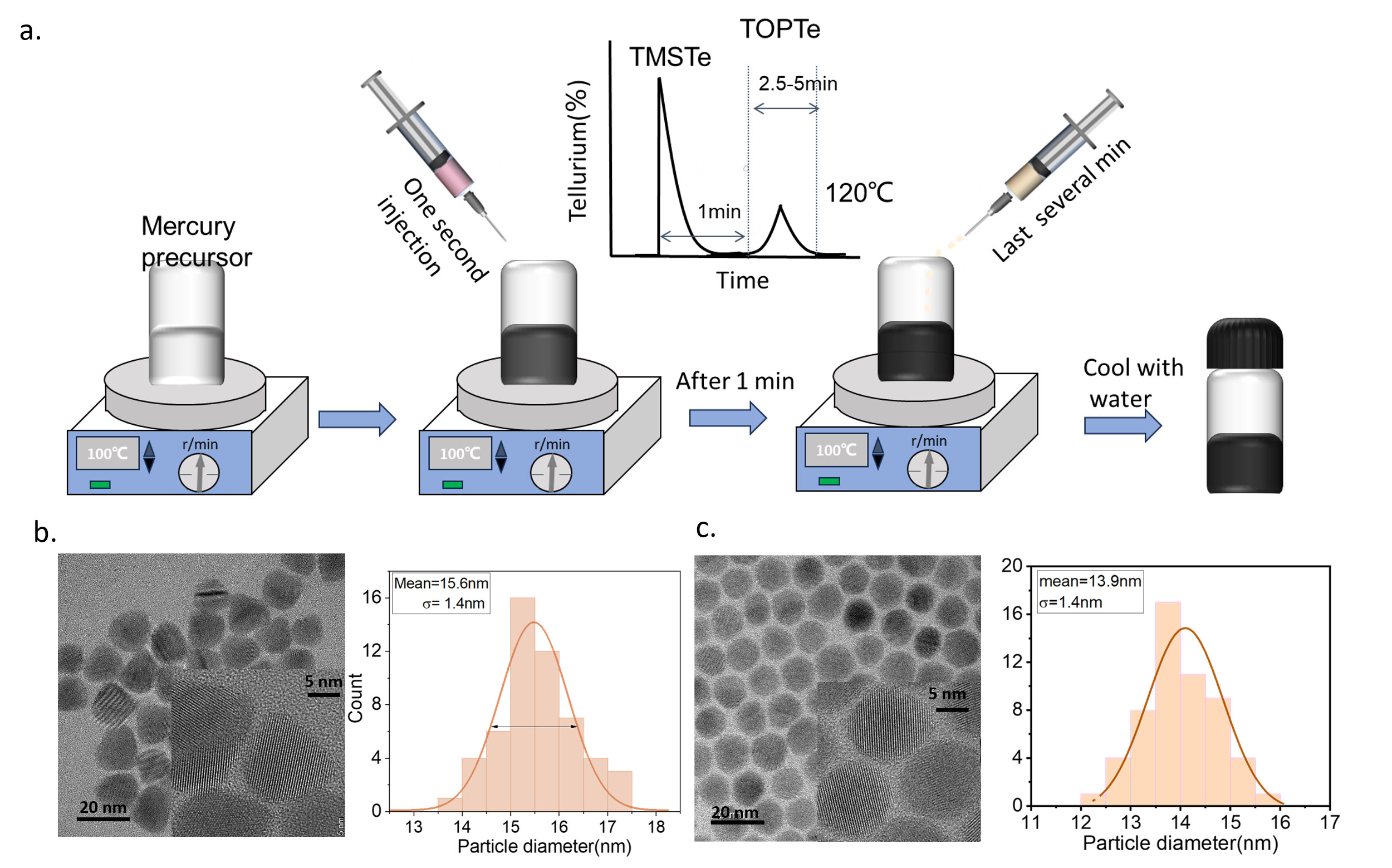


**Figure S1. Synthesis process and size distribution of VLWIR and LWIR CQD**. (a) Synthesis flow chart of VLWIR CQD and LWIR CQD. (b, c) TEM picture and size distribution histogram of VLWIR CQD and LWIR CQD, respectively.

1. **Mixed phase ligands exchange process**
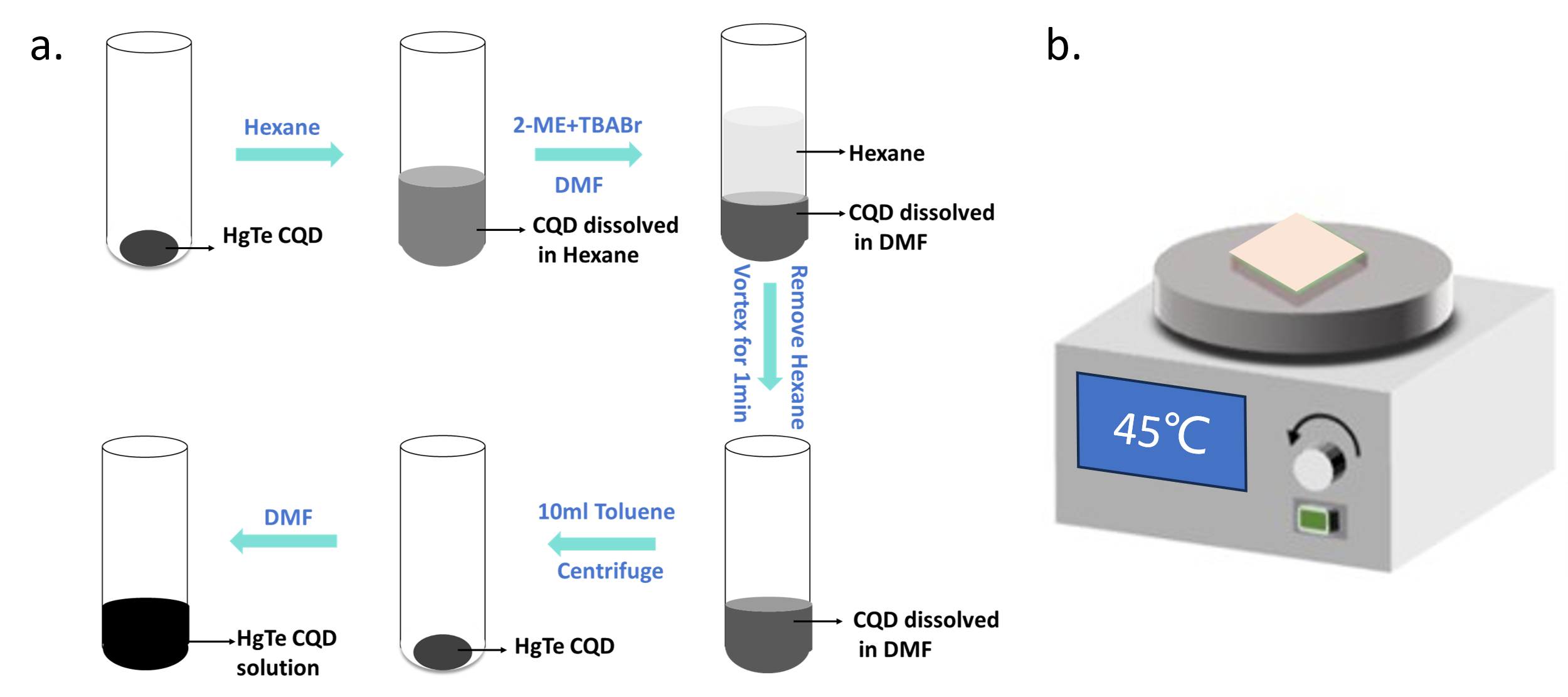
**.**

**Figure S2. CQD solid preparation**. (a) Mixed-phase ligand exchange process. (b) Schematic diagram of quantum dot spin-coating apparatus.

**Figure S2** illustrates mixed-phase ligand exchange process following the previous reference^1^. The solids are prepared by spray-coating. Polar phase transfer is used taking advantage of the difference in polarity between hexane and N,N-Dimethylformamide (DMF). The large size CQD could be stable in DMF for several months. To prepare high quality solids, toluene is added into CQD/DMF solution. After centrifuge, the precipitate would be dissolved in small amount of DMF for high concentration. The CQD films are spin-coated on substrates heated at 45℃, which is favorable for the high uniformity. All films would be treated with ethanedithiol (EDT)/HCL/ isopropanol (IPA) (1:1:50 by volume) and rinse with IPA.

1. **Iodine treatment.**

**
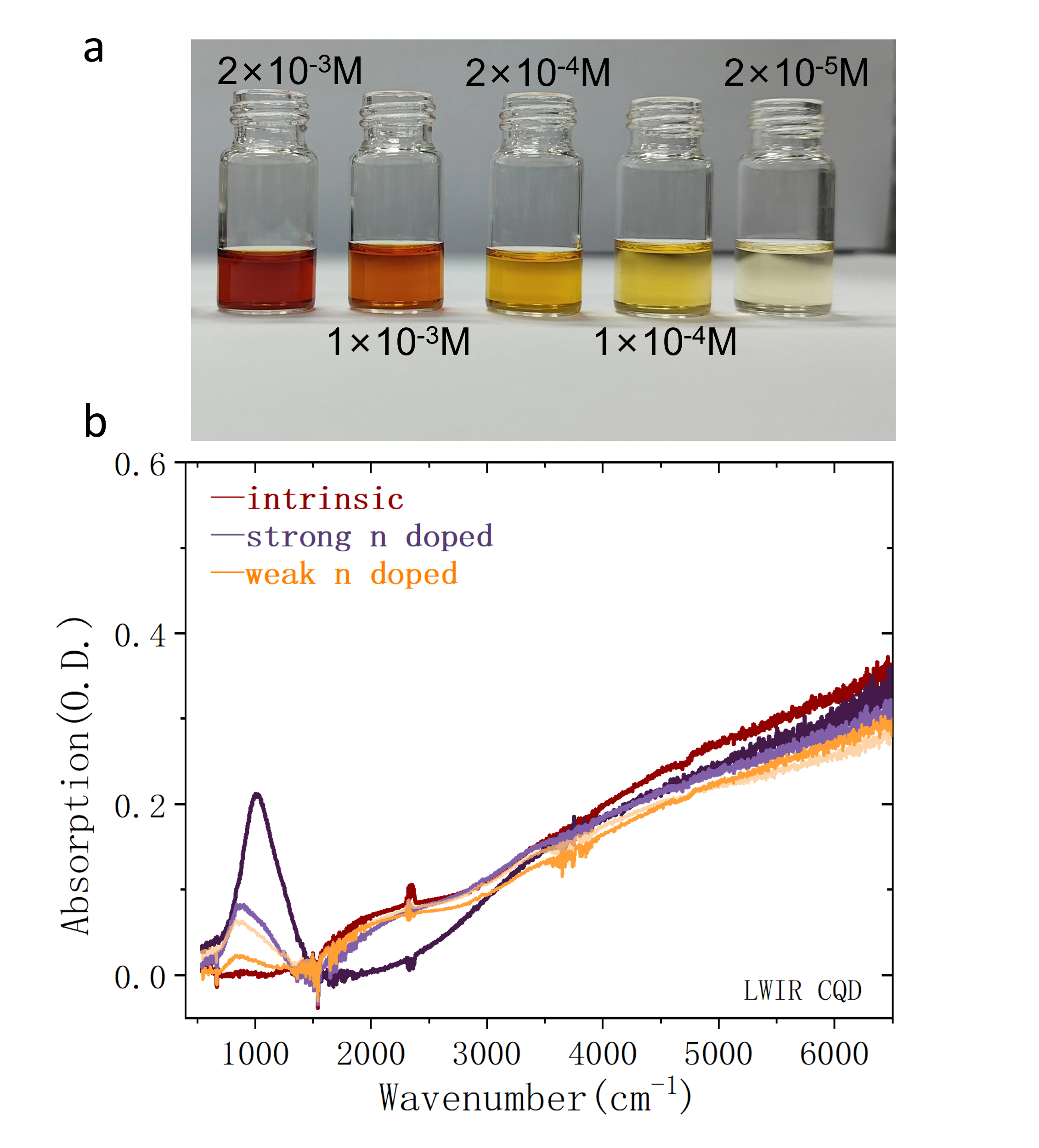
**

**Figure S3. Iodine treatment**. (a) Iodine/ethanol solutions with different concentrations. (b) Absorption spectra of films treated with different iodine concentrations

**Figure S3a** shows the picture on iodine/ethanol solutions with different concentrations. **Figure S3b** shows the absorption spectra of LWIR CQD films treated with different iodine concentrations and time. For as prepared CQD film, the absorption spectrum exhibits a strong N-type (purple line). Initially, a 2×10^-5^ M concentration iodine solution was drop-cast onto the as-prepared CQD film. After 30 seconds, the film was rinsed with IPA, dried with a nitrogen gun. The absorption spectrum (pale purple line) show reduced intraband absorption (reduced n type doping). With the increase in concentration, the intraband transitions gradually diminished. Using 2×10^-4^ M iodine solution for 1min, the intraband transitions completely disappeared (brown line).

Also, the resistance of the detector is measured by an ohmmeter, which gradually increase with increasing iodine time with 2×10^-4^ M solutions concentration. We stop the iodine treatment when the resistance starts to decrease, where the CQD film is nearly intrinsic and to turn p type. If one continuing to treatment time, the resistance becomes substantially smaller, indicating CQD shift to the P type. The higher iodine concentration above 1×10^-3^ M would cause the CQD less shiny, which may damage the solid.

1. **Photoluminescence.**

Currently, most commercial photoluminescence measurement setups only cover the short-wave infrared (2200 nm). To investigate our LWIR and VLWIR CQD, we modify the homemade set up as shown in **Figure S4**. The sample is excited with an 808 nm laser (LDM56/M) modulated at 100 kHz. The main part of the setup is Michelson interferometer with a single pixel MCT detector with spectral range 2-20 μm at 80 K. The Michelson interferometer is controlled by a step motor. The MCT detector output is sent to oscilloscope and phase-locked amplifiers simultaneously. As the interference pattern is observed through oscilloscope, the computer obtains the output signal *via* phase-locked amplifier and high-speed data acquisition card. The photoluminescence spectra would be obtained by Fast Fourier Transform (FFT).


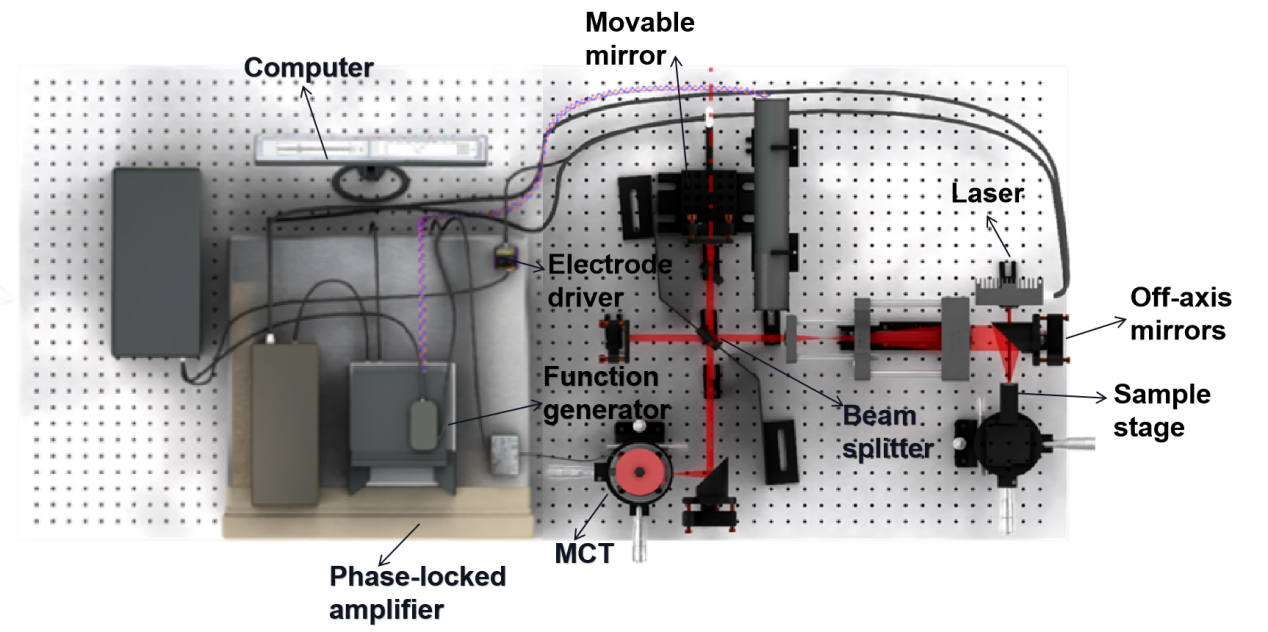


**Figure S4. homemade photoluminescence setup.**

1. **X-ray Diffraction.**

**Figure S5** shows the X-ray diffraction (XRD) result before and after iodine treatment. Obviously, the lattice structures are essentially identical before and after I_2_ treatment, which is *β*-HgTe (zinc-blende). The measurement is performed with the D8 ADVANCE X-ray diffractometer from Bruker, Germany. The instrument was operated with a tube current of 40mA and a tube voltage of 40kV. The Cu target had a wavelength of 1.5406 Å, while the Co target had a wavelength of 1.79026 Å.

**
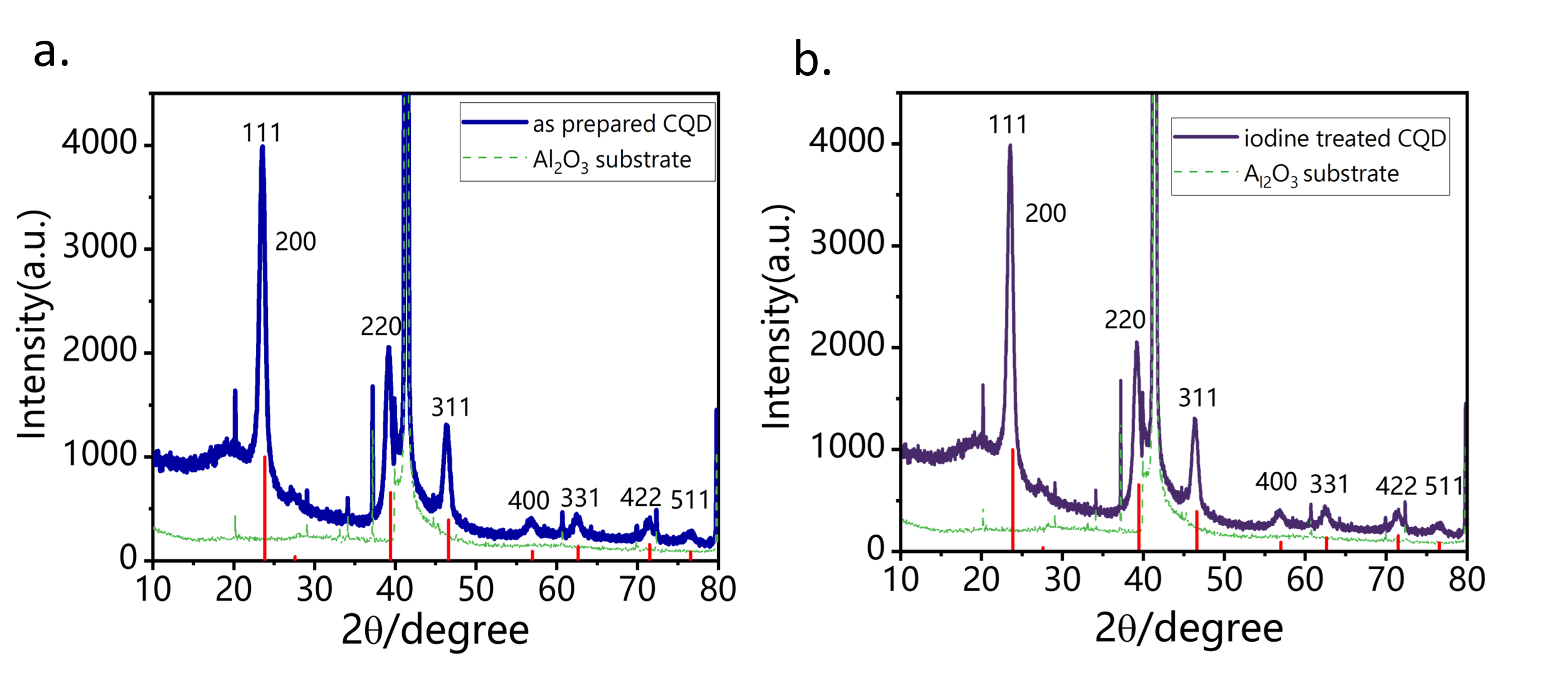
**

**Figure S5. XRD on CQD before and after iodine treatment.**

1. **X-ray Photoelectron Spectroscopy.**

The CQD solid surface before and after iodine treatment is analyzed by X-ray Photoelectron Spectroscopy (XPS), shown in **Figure S6,** with the element ratio table extracted from XPS analysis. The equipment is PHI QUANTERA-II SXM, by ULVAC-PHI Corporation, Japan. The X-ray source is AlKa (Al target, 1486.6 eV).


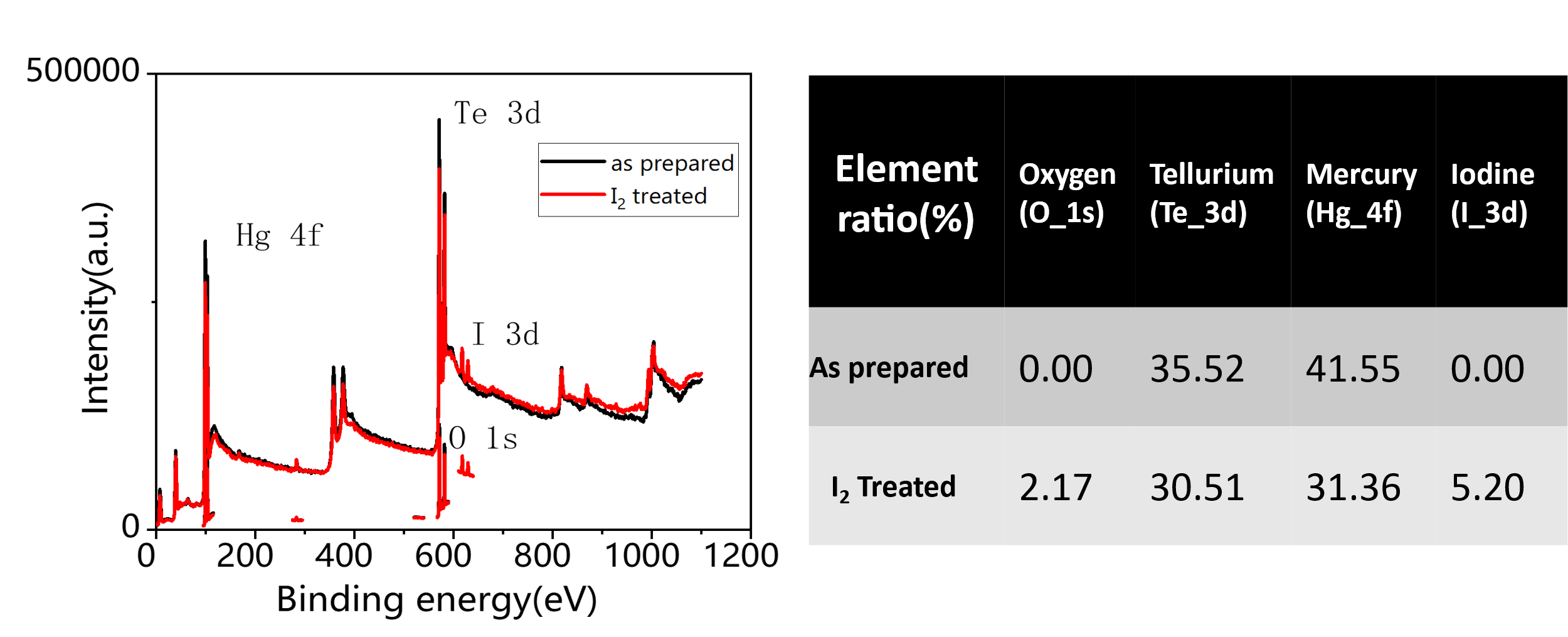


**Figure S6. XPS on CQD before and after iodine treatment.**

1. **Theoretical band calculations**

We used the DFT as implemented in the Vienna Ab initio simulation package (VASP) in all calculations. The exchange-correlation potential is described by using the generalized gradient approximation of Perdew-Burke-Ernzerhof (GGA-PBE). The projector augmented-wave (PAW) method is employed to treat interactions between ion cores and valence electrons. The plane-wave cutoff energy was fixed to 500 eV. Given structural models were relaxed until the Hellmann–Feynman forces smaller than -0.02 eV/Å and the change in energy smaller than 10^-5^ eV was attained. During the relaxation, the Brillouin zone was represented by a Γ centered k-point grid of 10×10×10. Grimme’s DFT-D3 methodology was used to describe the dispersion interactions among all the atoms in adsorption models. The CQD structures are showed in **Figure S7.** The 15.6 nm diameter CQD is tetrahedral while the 13.9 nm diameter CQD is spherical.


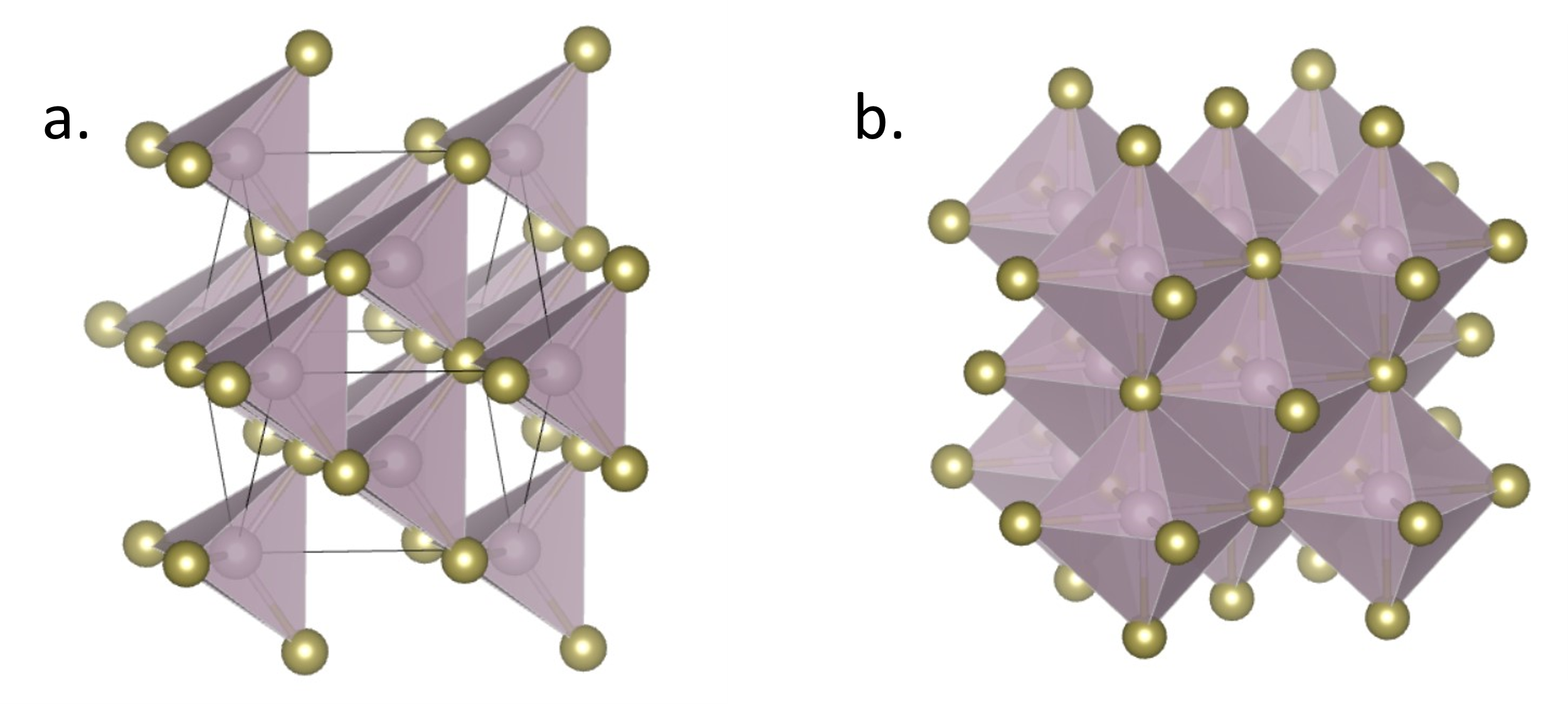


Figure S7. DFT with VASP. (a) Tetrahedral CQD. (b) Spherical CQD.

We notice the HgTe CQD band structure has special degeneracy, especially below the Fermi level. These degeneracies typically involve spin-orbit coupling and time-inversion symmetry. When the size of HgTe quantum dots changes, these degeneracies may be broken or rearranged.

1. **Typical transport characterization on VLWIR HgTe CQD.**


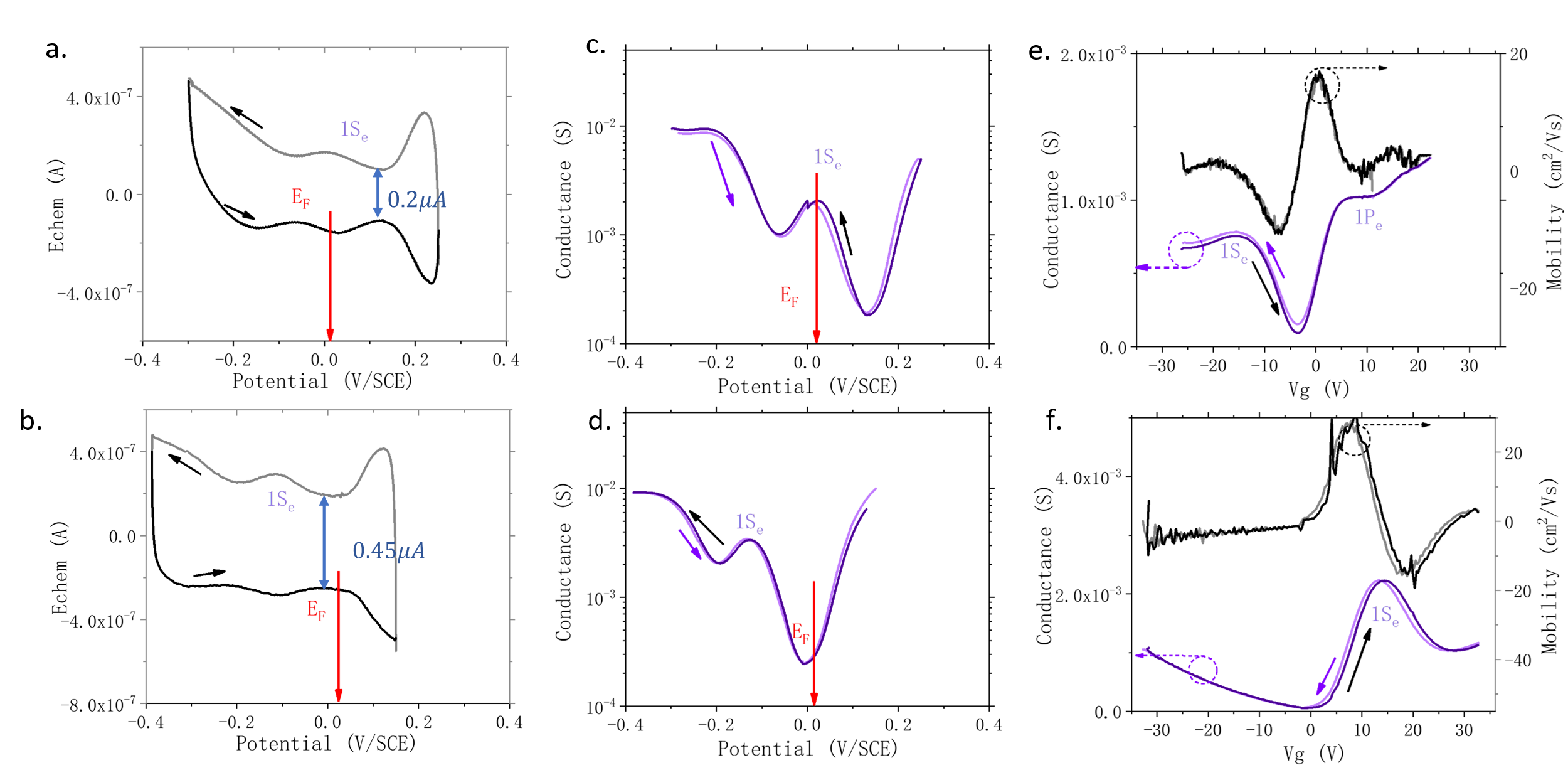


**Figure S8. Transport characterization on VLWIR CQD.** (a, b) Cyclic voltammetry on LWIR HgTe CQD before and after I_2_ treatment at 203 K, respectively. The arrows indicate forward and backward scan direction. Red arrows show the rest potential (Fermi Level) before the scan. (c, d) Conductance at different potential measured by electrochemistry with bi-potentiostat before and after I_2_ treatment, respectively. (e, f) FET transfer curve (purple lines) and differential mobility (black lines) at 80 K of HgTe CQD before and after I_2_ treatment, respectively.

Similar to LWIR CQD discussed in the main text, electrochemistry results show that as prepared VLWIR CQD solids have electrons doped in conduction band while the I_2_ treated CQD is near intrinsic, as shown in **Figure S8a-8b**, respectively. Fermi level E_F_ is noted in red. One could see the I_2_ treatment show a small shift on Fermi level, and negative shift on CQD energy band. 5mV bias difference is applied between the working electrodes where we obtain the conductance as a function of state density, as showed in **Figure S8c-8d**. Both cyclic voltammetry and conductance results show the quantum confined electronic states, noted as 1Se. The 1Pe state is less obvious compared to LWIR CQD, might due to the dense energy states.

Additionally, we also find increased reversible voltammetry currents on I_2_ treated CQD compared with the as prepared CQD solid, which may come from the redox process of I_2_/I_3_^-^/I^-^.

For FET measurement, silicon wafers with dry thermal oxide (n^++^ Si/300 nm SiO_2_) are used as the substrates. The drift mobility is extracted in the linear regime, calculated by fitting the transfer curve following equation: $\mu^{FET} =$ $\frac{L}{WC_{i}V_{D}}\frac{dI_{D}}{dV_{g}}$ , where *L*, *W*, *C*_i_, *V*_D_, *I*_D_, and *V*_g_ are the channel length, channel width, capacitance per unit area, drain voltage, drain current, and gate voltage, respectively. We also verified that in all measurements FET channel current exceeded the gate leakage current by several orders of magnitude. The electrode design is mentioned in the main text as the photoconductor where 25 pairs of interdigitated evaporated gold electrodes with finger width 10 microns, gap 10 microns (channel length in FET), and finger length 1mm (channel width in FET). For 300 nm SiO_2_, $C_{i}$=1.15x10^-4^ F/m^2^. Carrier density is estimated by $\Delta V_{g}C_{i}/N$, where $\Delta V_{g}$ is the absolute gate potential where the film conductivity reaching minimum in FET measurement result, N the CQDs areal density ($\propto d^{-2}$, $d$ the CQD diameter). FET transfer curve and mobility are shown in **Figure S8e-8f.**

1. **Electrochemistry on iodine solution.**

**Figure S9** shows the cyclic voltammetry measurement on 0.001M I_2_/ethanol. The arrow shows the scan direction. From the cyclic voltammetry, iodine oxidation clearly occurs in two steps, attributing to the I_2_/I_3_^–^ and I_3_^–^/ I^–^ processes at lower and higher potentials. The increased voltammetry currents mean more external carrier injection or depletion is needed to tune doping level, which is beneficial for doping stabilization in these CQD solids. The band gap of HgI_2_ is wide, 2.13eV, which might be an advantage compared with previous S^2-^ treatment where narrow gap HgS would exist. We note that there are contaminants like water and oxygen in ethanol solution. That may explain the small ratio of oxygen element on CQD surface after I_2_ treatment.


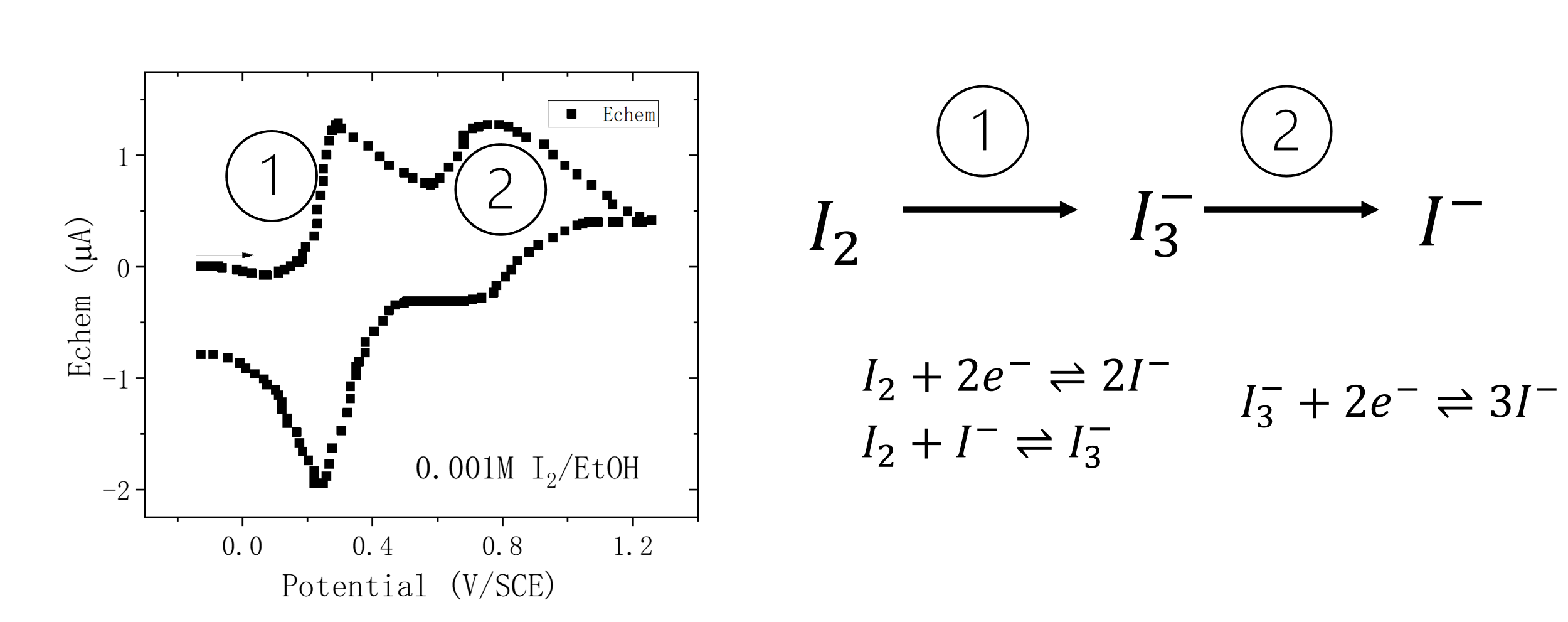


**Figure S9. Electrochemistry on 0.001M I_2_/ethanol.**

1. **Transport on CQD without mixed phase ligands exchange.**


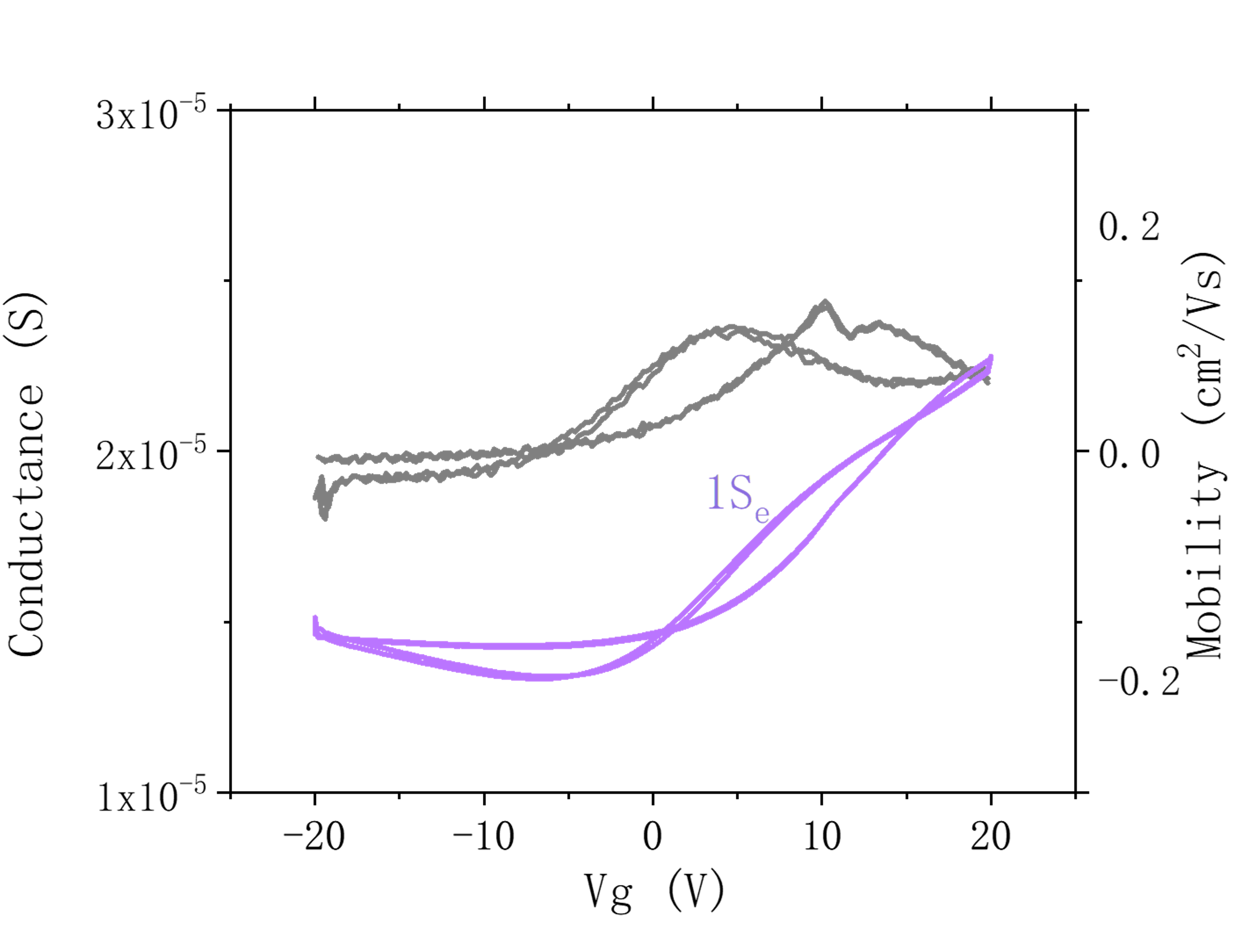


**Figure S10. Low mobility VLWIR CQD.** FET transfer curve (purple lines) and differential mobility (black lines) at 80 K of low mobility HgTe CQD after I_2_ treatment.

**Figure S10** show the field effect transistor measurement on low mobility VLWIR HgTe CQD solid at 80 K, where the mobility is ~0.1 cm^2^/Vs. This low mobility CQD solid is prepared by CQD dissolved in hexane, whose surface ligands should be OAm. Then, the solid ligands exchange is used with EDT/HCl/IPA for 30 sec. The hysteresis is much larger than high mobility CQD solids. Still, one could figure out quantum confined 1Se state on transfer curve.

1. **Mobility as a function of temperature.**
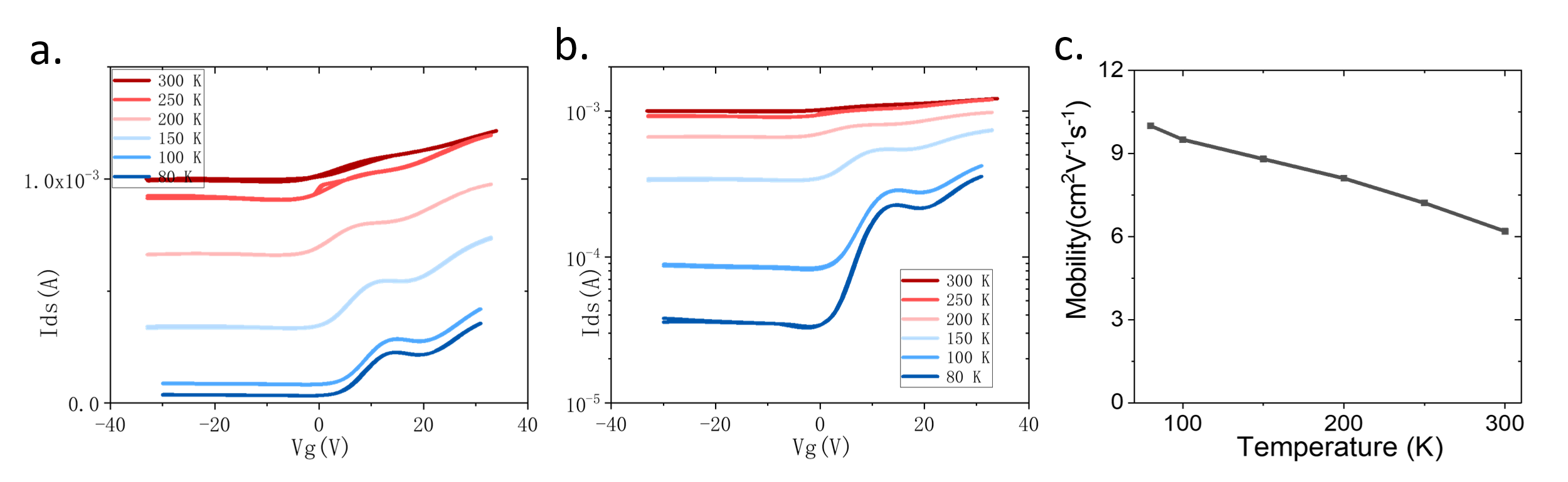


**Figure S11. Mobility as a function of temperature.** (a, b) FET transfer curve on VLWIR CQD at different temperatures in linear and log scale. (c) Mobility as a function of temperature extracted from FET transfer cureves.

1. **The spectral response with wavenumber as the x-axe.**


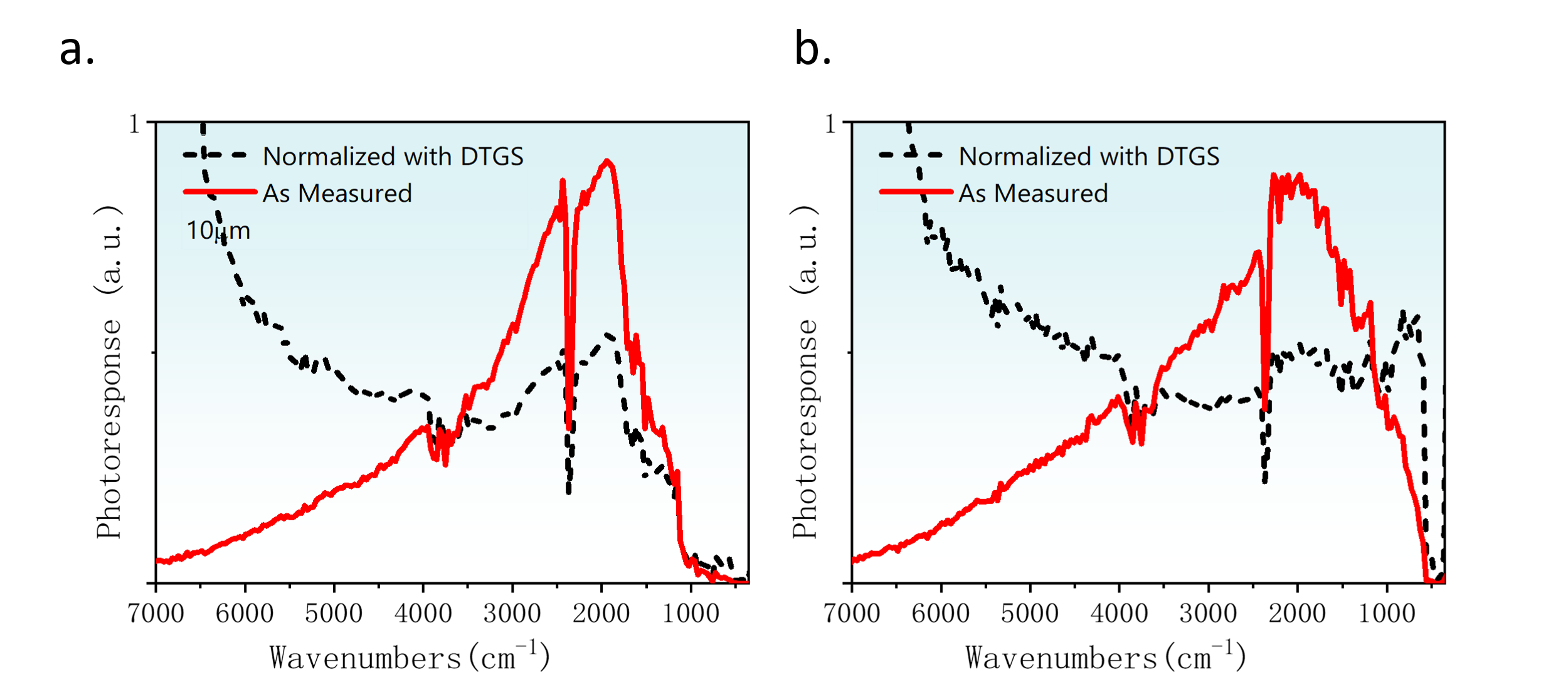


**Figure S12. The spectral response with wavenumber as the x-axe.** (a) LWIR CQD photodetector. (b) VLWIR CQD photodetector.

1. **The spectral response on LWIR CQD at different temperatures.**


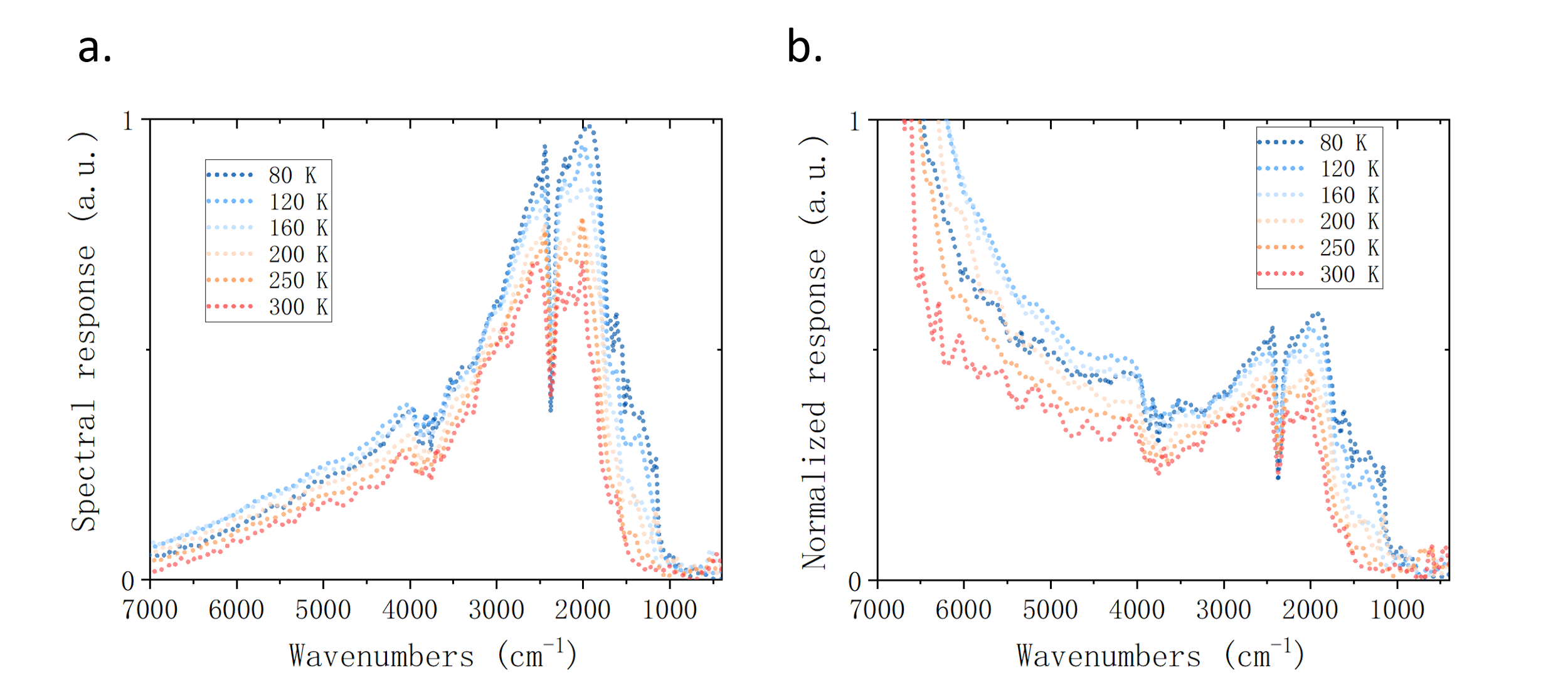


**Figure S13. The spectral response on LWIR CQD at different temperatures.** (a) as measured spectral response. (b) normalized spectral response.

For LWIR, we successfully measure the spectral response from 300 K to 80 K as shown in **Figure S13**, where the room temperature spectral response edge is 1720 cm^-1^ (5.9 μm), comparable with the room temperature absorption edge 1600 cm^-1^ (6.3 μm).

For VLWIR, although there is a small photocurrent at room temperature, we could not get the spectral response due to the high dark current and noise density.

1. **The spectral response comparison.**


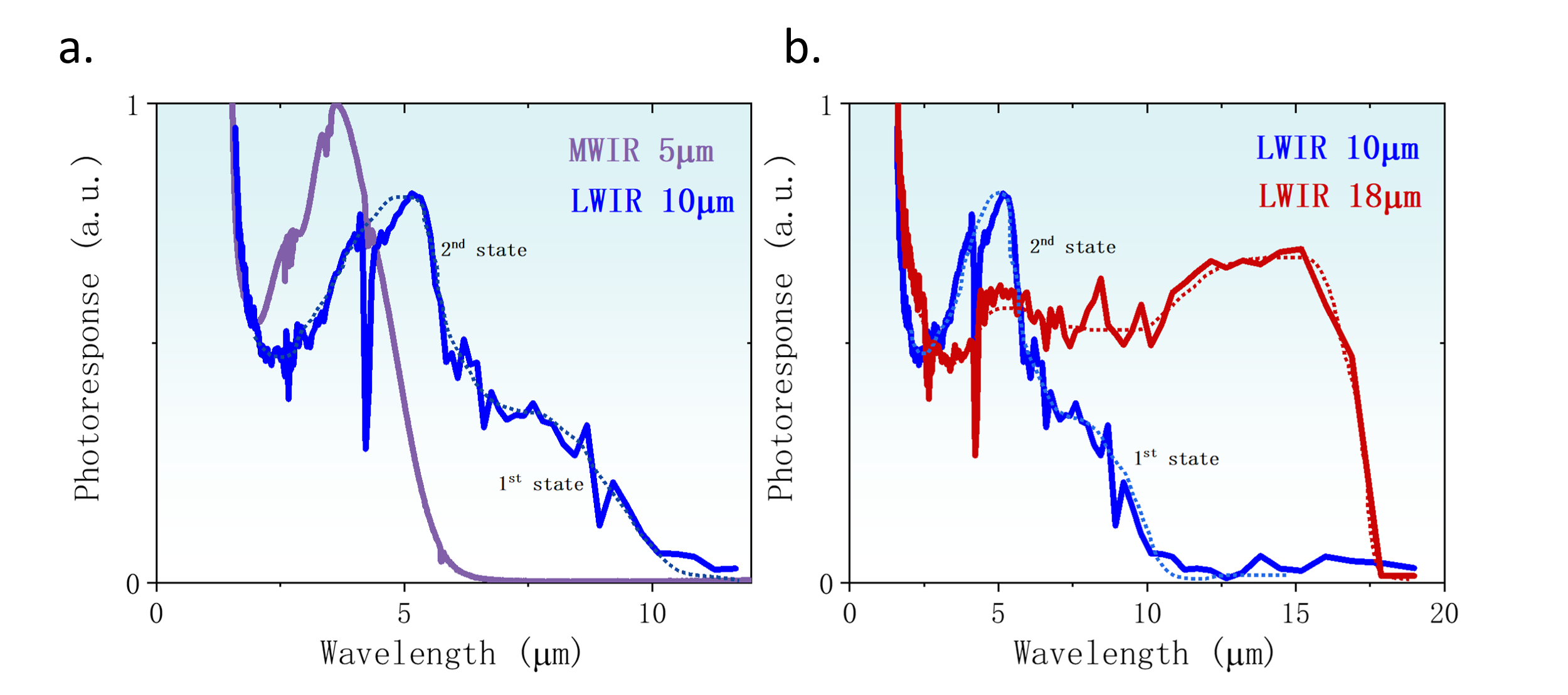


**Figure S14. The spectral response comparison among MWIR, LWIR and VLWIR CQD Photodetectors.**

**Figure S14** shows the spectral response comparison among MWIR, LWIR and VLWIR CQD Photodetectors. In MWIR and LWIR, the quantum confined energy states are configurable at noted as the 1^st^ and 2^nd^ states. For VLWIR CQD photodetector, the states are unconfigurable due to the dense states, which is like bulk semiconductors.

1. **Absorption coefficient calculation.**

Measured transmission spectra on 500 nm LWIR CQD on ZnSe window is showed in **Figure S15a**. The absorption coefficient$\alpha\left( \lambda\right)=\frac{4\pi k}{\lambda}=-\frac{1}{z}ln(T(\lambda))$, $T(\lambda)$ is the transmission, z=500 nm is the solid thickness^2^. We also give the absorption coefficient values on LWIR, MWIR, Extend SWIR (2.5 μm) and SWIR (2 μm) CQD are ~800 cm^-1^, 10000 cm^-1^, 6000 cm^-1^ and 6000 cm^-1^, respectively, for a more obvious comparison. It is surprising to see the one order drop in the absorption coefficient values on LWIR CQD, compared to shorter wavelength. This may come from the sparse hole density as well as the large size of the LWIR CQDs.


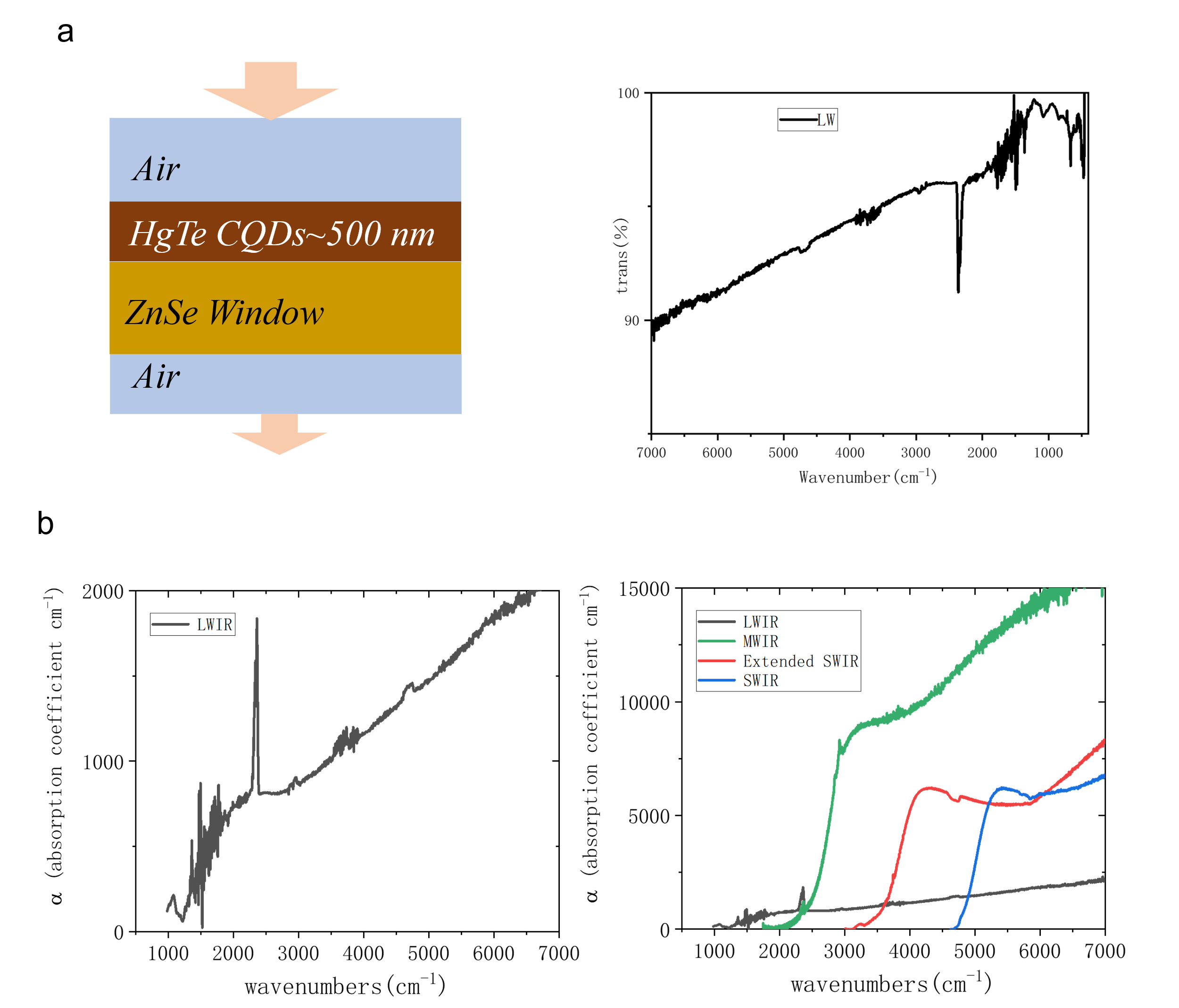


**Figure S15. Absorption coefficient.** (a) Measured transmission spectra on 500nm LWIR CQD on ZnSe window. (b) Calculated absorption coefficient on LWIR, MWIR, extended SWIR and SWIR, respectively.

When light propagates a distance z through a medium with a refractive index n+ik containing N particles per unit volume, the intensity is reduced by exp(-C_ex_Nz). C_ex_ is the extinction cross section, which is the sum of the absorption cross sections (C_abs_) and scattering cross sections (C_scat_). For particles with radius (a) much smaller than the wavelength in the medium, C_abs_ is the main term. The absorption coefficient α=C_ex_N= C_abs_N.

The absorption coefficient directly related to particle size. For LWIR CQD, N=2.6×10^17^cm^-3^. With α=800 cm^-1^, C_abs_=3×10^-15^cm^2^. This value is agreed with the reference that the band edge particle cross section is typically around 1.5 **×** 10^−15^ cm^2^.^3^

1. **Dark current at different temperatures.**


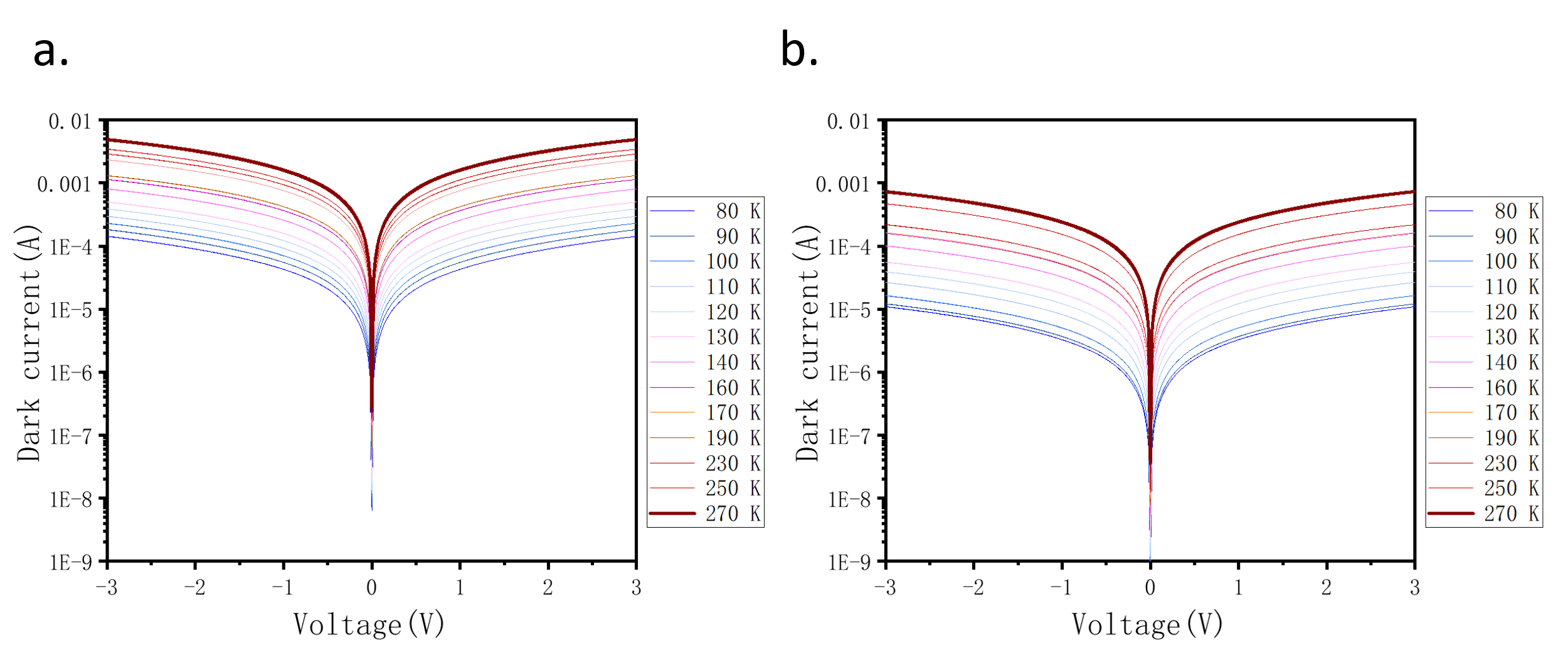


**Figure S16. Dark current at different temperatures.** (a, b) Dark currents as a function of applied bias at different temperatures on VLWIR and LWIR CQD photoconductor, respectively.

1. **Gaussian fittings on spectrum**

The absorbance spectrum for 15.6 nm HgTe CQD can be fit with a sum of Gaussians and a parabolic background. As shown in Figure R3, the data is shown as a bold black line, shown in red. The inset demonstrates the corresponding electronic transitions. For 2-10 μm range, the blue, orange, and yellow Gaussian peaks respectively represent the interband absorption peaks for 1S_h_-1S_e_, 2S_h_-1S_e_ and 1S_h_-1P_e._ The gradual broadening of the Gaussian peak is due to the merging of neighboring energy levels. We only show obvious absorption peaks from the spectrum, and other higher-energy interband transition peaks also were merged to show broader peaks. Within the 10-20 μm range, the green, purple, and pink Gaussian peaks respectively represent the intraband transitions of 1S_e_-1P_e_^1/2^, 1S_e_-1P_e_^3/2^. The gray shadow is the parabolic background from the scattering of the CQD film (~Ax^2^ (x is the wavenumber)), scattering is enhanced as the wavenumber increases.


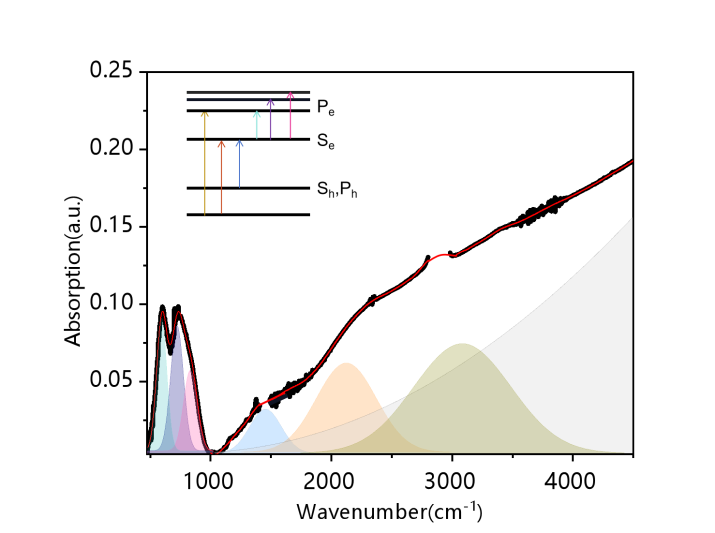


**Figure S17.** **Gaussian fittings on spectrum**

1. **Detectivity comparison**

We compare the typical LWIR/VLWIR photodetectors in Table S1.

**
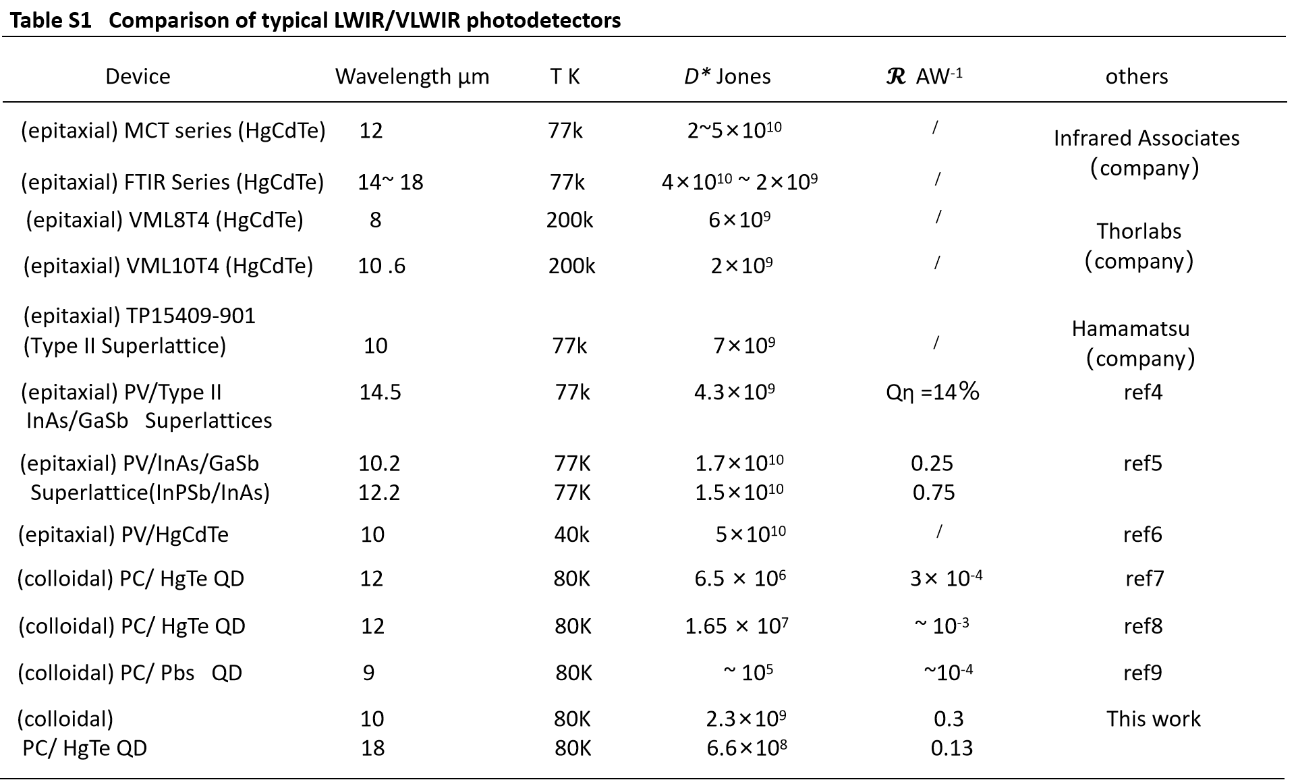
**

1. **Temporal response**

The schematic diagram of the response speed measurement is shown in Figure S18a. The wavelength of laser used for the response speed measurements is 1550 nm. The light emitted by the laser fiber enters the polarization controller, which controls the polarization state of the light. Then, the light entering the electro-optical modulator is modulated into a pulsed laser signal. The signal waveform generator is connected to the electro-optical modulator, adjusting the parameters such as frequency and amplitude of laser signal. Meanwhile, the signal waveform generator is connected to the oscilloscope to observe the modulated signal, which is then incident on the photodetector.

The photodetector is connected in series with a bias voltage and a resistor that matches the internal resistance value of the detector. The time constant rise ($\tau_{\mathrm{rise}}$) refer to the time required for the signal voltage to rise from 10% of the maximum value to 90%. The response speed of LWIR and VLWIR detectors are shown in Figure S18 b-c. The $\tau_{\mathrm{rise}}$ are 93.4 μs and 368 μs respectively.


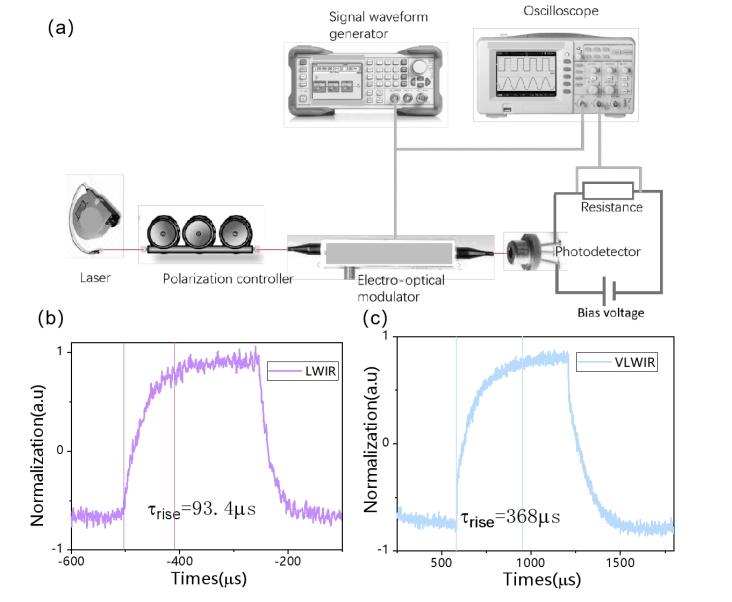


**Figure S18.** **Temporal response**

1. **Transport property summary**

We list a table on transport property to give a clear comparison on high and low mobility CQD.

**
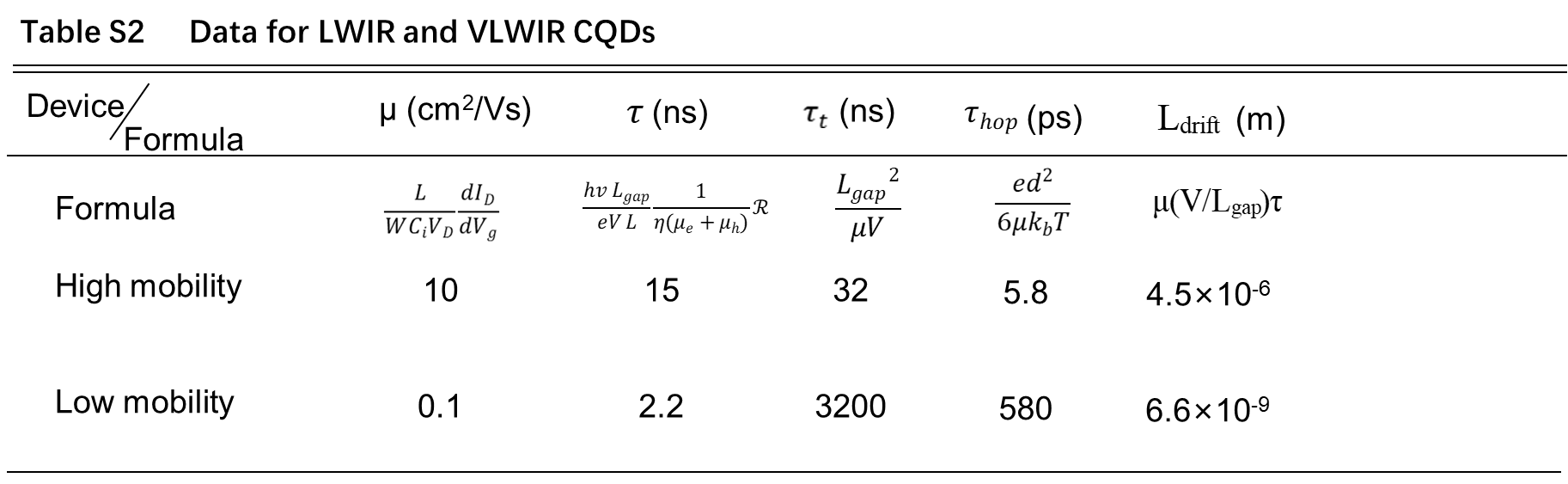
**

1. **Spectral Detectivity**

We calculate the spectral detectivity of the VLWIR detector as shown in Figure S19. By the formula $B\int R\left( \tilde{v} \right)I\left( \tilde{v} \right)d\tilde{v}=I_{\mathrm{ph}}$, $R\left( \tilde{v} \right)$ is the relative spectral response of the detector after normalization, $I\left( \tilde{v} \right)$is the blackbody radiation curve of 600℃, multiply $I\left( \tilde{v} \right)$ with $R\left( \tilde{v} \right)$ and integrals.

The coefficient *B* can be obtained, which is 0.47. The absolute spectral response of the detector can be obtained by multiplying *B* by $R\left( \tilde{v} \right)$.The spectral detectivity D*=$\frac{B *R\left( \tilde{v} \right) \sqrt{A_{d}}}{I_{n}}$,$A_{d}$is the detector area, $I_{n}$ is the noise, which the peak detectivity is 1×10^9^ Jones.


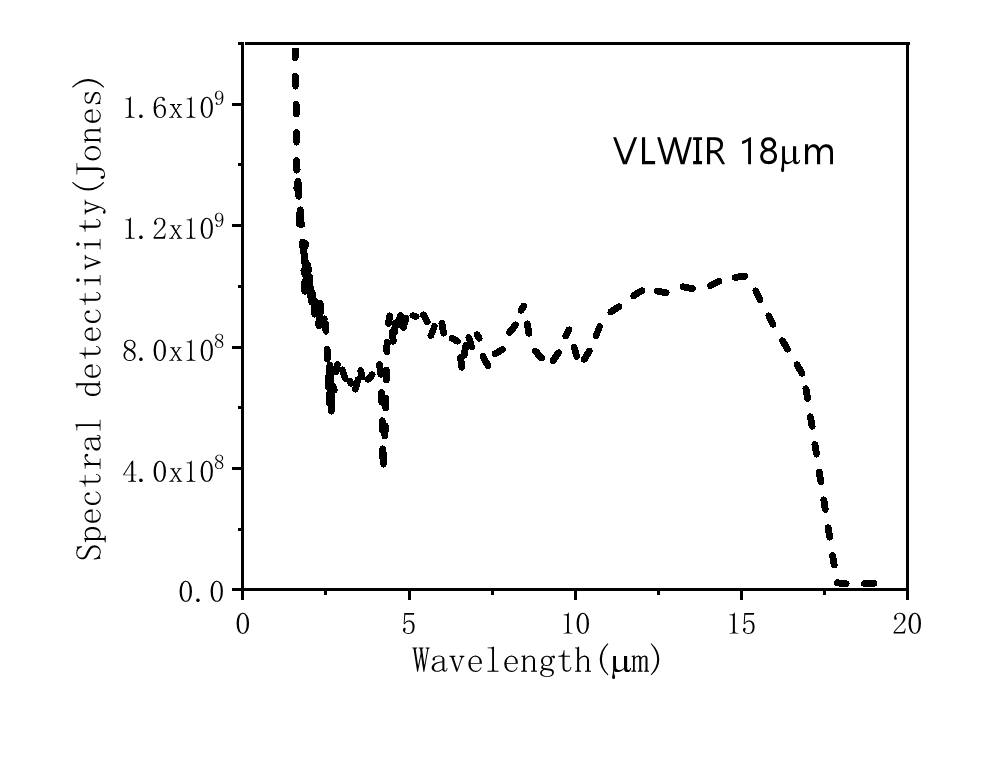


**Figure S19. Spectral Detectivity**

**Reference**

1. Chen, M., Hao, Q., Luo, Y. & Tang, X. Mid-Infrared Intraband Photodetector via High Carrier Mobility HgSe Colloidal Quantum Dots. *ACS Nano* **16**, 11027-11035 (2022).
2. Xue, X. et al. High-operating-temperature mid-infrared photodetectors via quantum dot gradient homojunction. *Light Sci. Appl.* **12**, 2 (2023).
3. Lhuillier, E., Keuleyan, S. & Guyot-Sionnest, P. Optical properties of HgTe colloidal quantum dots. *Nanotechnology* **23**, 175705 (2014).
4. Wei, Y. et al. High Structural Quality of Type II InAs/GaSb Superlattices for Very Long Wavelength Infrared Detection by Interface Control. *IEEE Journal of Quantum Electronics* **48**, 512-515 (2012).
5. Liu, J. et al. Huang, Y., Long-wavelength InAs/GaSb superlattice double heterojunction infrared detectors using InPSb/InAs superlattice hole barrier. *Semiconductor Science and Technology* **37** (2022).
6. Velicu, S. et al. Wijewarnasuriya, P., MWIR and LWIR HgCdTe Infrared Detectors Operated with Reduced Cooling Requirements. *Journal of Electronic Materials* **39**, 873-881 (2010**)**.
7. Keuleyan, S. E., Guyot-Sionnest, P., Delerue, C. & Allan, G. Mercury telluride colloidal quantum dots: electronic structure, size-dependent spectra, and photocurrent detection up to 12 μm. *ACS Nano* **8**, 8676-8682 (2014)
8. Zhang, H., Peterson, J. C., Guyot-Sionnest, P. Intraband transition of HgTe nanocrystals for Long-Wave infrared detection at 12 μm. *ACS Nano* **17**, 7530-7538 (2023)
9. Ramiro, I. et al. Mid- and Long-Wave infrared optoelectronics via intraband transitions in PbS colloidal quantum dots. *Nano Lett*. **20**, 1003-1008 (2020).
